# Understanding contraceptive switching rationales from real world clinical notes using large language models

**DOI:** 10.1038/s41746-025-01615-0

**Published:** 2025-04-23

**Authors:** Brenda Y. Miao, Christopher Y. K. Williams, Ebenezer Chinedu-Eneh, Travis Zack, Emily Alsentzer, Atul J. Butte, Irene Y. Chen

**Affiliations:** 1 Bakar Computational Health Sciences Institute, University of California San Francisco, San Francisco, CA USA; 2 Department of Medicine, University of California San Francisco, San Francisco, CA USA; 3 Helen Diller Family Comprehensive Cancer Center, University of California San Francisco, San Francisco, CA USA; 4 Division of General Internal Medicine, Brigham and Women’s Hospital, Boston, MA USA; 5 Harvard Medical School, Boston, MA USA; 6 Center for Data-driven Insights and Innovation, University of California, Office of the President, Oakland, CA USA; 7 Computational Precision Health, University of California, Berkeley and University of California, San Francisco, Berkeley, CA USA; 8 Electrical Engineering and Computer Science, University of California, Berkeley, Berkeley, CA USA; 9 Berkeley AI Research, University of California, Berkeley, Berkeley, CA USA

**Keywords:** Medical research, Risk factors, Public health

## Abstract

Understanding reasons for treatment switching is of significant medical interest, but these factors are often only found in unstructured clinical notes and can be difficult to extract. We evaluated the zero-shot abilities of GPT-4 and eight other open-source large language models (LLMs) to extract contraceptive switching information from 1964 clinical notes derived from the UCSF Information Commons dataset. GPT-4 extracted the contraceptives started and stopped at each switch with microF1 scores of 0.85 and 0.88, respectively, compared to 0.81 and 0.88 for the best open-source model. When evaluated by clinical experts, GPT-4 extracted reasons for switching with an accuracy of 91.4% (2.2% hallucination rate). Transformer-based topic modeling identified patient preference, adverse events, and insurance coverage as key reasons. These findings demonstrate the value of LLMs in identifying complex treatment factors and provide insights into reasons for contraceptive switching in real-world settings.

## Introduction

Prescription contraceptives play a critical role in supporting women’s reproductive health and patients may switch between several contraceptives throughout their health trajectories^1–3^. With many contraceptive options available, understanding the factors driving selection and switching can provide data to inform patient-provider decision making. Contraceptives may vary by active ingredient^4^ with each contraceptive group producing unique adverse event profiles that may contribute to clinical decision making^2,5^. In addition, several other factors, including personal preference, cost, availability, comorbidities and clinical constraints, may contribute to a patient’s decision to start, stop, or switch contraceptives^6^. With nearly 50 million women in the United States using contraceptives^7^, understanding the factors that drive contraceptives selection and switching is of significant interest^4,7,8^.

After a medication is prescribed, patients may elect to switch treatments for reasons related to efficacy, side effects, costs, access, or personal preference^9–12^. Contraceptive switching is common - 44% of women starting a contraceptive discontinued its use within 1 year, with 76% resuming use of the same or another contraceptive within 3 months^13^. However, the reasons behind treatment switches are often documented only in clinical notes, making them difficult to analyze at scale. Manual annotation to create datasets is time-consuming and expensive^14–16^, particularly for complex clinical text, and the development of machine learning models to automate this information extraction remains a challenging task^17^.

Recently, the development of general large language models (LLMs) has shown significant promise in being able to extract medication information without the need for manually annotated training data (“zero-shot extraction”)^18–20^. Despite concerns including factually incorrect information, clinicians and researchers remain optimistic that these computational advances can translate to clinically-meaningful use cases^21–24^. Here, we evaluate the ability of GPT-4 to extract reasons for contraceptive selection strategies. These extracted values were used to understand differences in reasons for switching between patient populations using clinical notes from a large academic medical center.

## Results

### Patient cohort

**Fig. 1:**
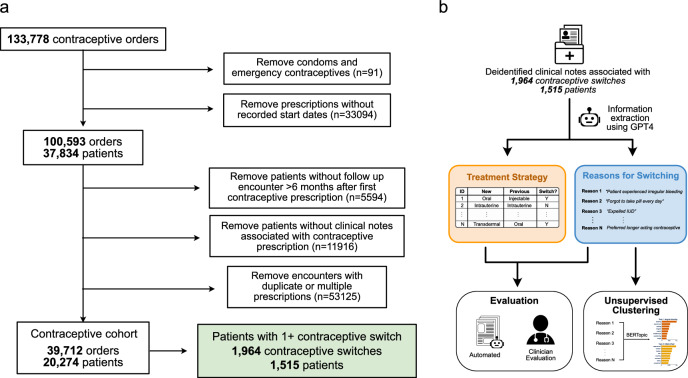
Study overview. **a** We selected a contraceptive patient cohort from the UCSF Information Commons dataset. Among 20,274 patients with unique contraceptive prescriptions and associated clinical notes, 1515 (7.6%) patients experienced a total of 1964 total contraceptive switches. **b** Study overview to assess the ability for GPT4 to extract contraceptive switching values from clinical notes, and to identify key reasons for switching using unsupervised clustering methods.

We selected a contraceptive patient cohort using the UCSF Information Commons dataset^25^, which contained 133,778 documented medication orders for contraceptives. Condoms and emergency contraceptives orders were removed, as were any prescriptions without start dates. The remaining cohort of 37,834 patients had a total of 100,593 relevant medication orders for an intrauterine, oral, intravaginal, subdermal, transdermal, or injectable contraceptive. We removed 5594 patients who did not have any follow up encounters at least 6 months after the last contraceptive order and further filtered out 11,916 orders without associated clinical notes. Finally, we removed 53,125 duplicate medication orders, leaving a contraceptive cohort consisting of 39,712 medication orders across 20,274 unique patients (Fig. 1a).

Among this contraceptive cohort, 1515 (7.6%) patients experienced a total of 1964 contraceptive switches. Compared to patients who did not have a contraceptive switch and had demographic information available (*n* = 15,907), patients with contraceptive switches tended to be younger, with a mean age of 25.9 years (SD: 7.7) compared to 29.1 years (SD: 8.4, *p* < 0.001, Table 1). The mean time to a patient’s first contraceptive switch was 39.1 months (SD: 32.0 months). There was also a statistically significant difference in the proportion of patients with and without contraceptive switches by patient race/ethnicity (*p* < 0.001). The largest difference occurred in patients with a race/ethnicity listed as “Black or African American,” with 19.3% of such patients having a contraceptive switch compared to 8.2% without. There was also a higher proportion of patients with a contraceptive switch identifying as “Latinx” (19.3%) compared to the proportion of “Latinx” patients without contraceptive switches (15.1%). “White” (33.0%) or “Asian” (16.0%) patients had lower rates of contraceptive switching in this cohort compared to the same groups without switches, with 45.1% of patients without contraceptive switches identifying as “White” and 20.3% identifying as “Asian.”

**Table 1:** Contraceptive prescription cohort demographics

	Contraceptive switch (*n* = 1515)	No switch (*n* = 15,907)	Significance Proportion
**Mean age (SD)**	25.9 years (7.7)	29.1 years (8.4)	***p*** < **0.001**
**Race/Ethnicity (%)**	Missing (*n* = 32)	Missing (*n* = 815)	***p*** < **0.001**
White	490 (33.0%)	6813 (45.1%)	
Latinx	286 (19.3%)	2281 (15.1%)	
Black or African American	286 (19.3%)	1237 (8.2%)	
Asian	237 (16.0%)	3071 (20.3%)	
Other	115 (7.8%)	1224 (8.1%)	
Multi-Race/Ethnicity	69 (4.7%)	466 (3.1%)	
**Preferred Language (%)**		Missing (*n* = 5)	***p*** < **0.001**
English	1474 (97.3%)	15405 (96.9%)	
Spanish	14 (0.9%)	281 (1.8%)	
Other	27 (1.8%)	216 (1.4%)	
**First prescribed contraceptive, (%)**			***p*** < **0.001**
Implant	160 (10.6)	799 (5.0)	20.0%
Injectable	199 (13.1)	853 (5.4)	23.3%
Intrauterine	64 (4.2)	1266 (8.0)	5.1%
Intravaginal	244 (16.1)	1935 (12.2)	12.6%
Oral	661 (43.6)	10496 (66.0)	6.3%
Transdermal	187 (12.3)	558 (3.5)	33.5%

Demographic information from all patients with contraceptive medication prescriptions. Patients are stratified into groups with and without contraceptive modality switching. Significance is reported between patients with and without contraceptive switching.

Switching differed significantly by the first contraceptive prescribed, with the highest rates of switching following initial prescription of transdermal contraceptives (33.5%) and the lowest rates following initial prescription of intrauterine (5.1%) and oral (6.3%) contraceptives. The most common switch occurred in patients who were on oral contraceptives and switched to intravaginal contraceptives (*n* = 205, *n* = 10.5%). The least common switch occurred from intrauterine to injectable contraceptives (*n* = 6, 0.31%, Supplementary Table 4).

### Human evaluation of GPT4 extraction of contraceptive switching

**Fig. 2:**
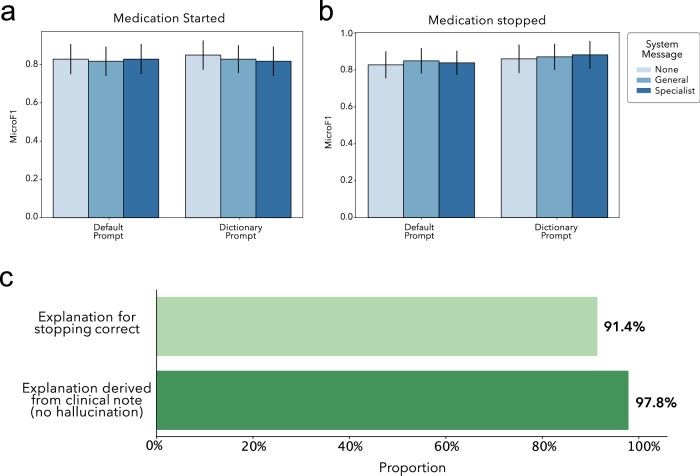
Development of prompt to extract contraceptive switching information. GPT4-extracted values for contraceptive class (**a**) started and **b** stopped compared to human annotation (*n* = 93). **c** Human evaluation was also performed to assess whether GPT-4 extracted reasons for contraceptive switching was accurate, and contained only information specifically mentioned in the associated clinical note (not hallucination).

Prompt evaluation was performed on a held out set consisting of notes from 5% of patients (*n* = 93 clinical notes), and evaluated against annotations from a clinical reviewer (Fig. 1b). There was no significant difference in performance across the six prompts used to extract contraceptive information using zero-shot GPT-4, with micro F1 scores ranging from 0.817 to 0.849 (mean = 0.827, SD: 0.012) for extraction of contraceptive started, and 0.827 to 0.881 (mean = 0.854, SD: 0.020) for extraction of contraceptive stopped (Fig. 2a, b). The best prompt for medication stopping extraction used the specialist system configuration and default prompt. Reasons extracted by this prompt were also evaluated by a clinical reviewer for both accuracy and rate of hallucination. Human evaluation showed that GPT-4 was capable of extracting these reasons with 91.4% accuracy and without hallucination 97.8% of the time (*n* = 93, Fig. 2c). Given the high accuracy and minimal hallucination of this prompt for extracting information about contraceptive stopping and reasons for stopping on the development dataset, this prompt was selected to extract contraceptive information from the remaining clinical notes.

The performance of this prompt was also tested in several open source language models (Gemma-7B-it^26^, Gemma2-9B-it^27^, Meta-Llama-3-8B-Instruct^28,29^, Meta-Llama-3.1-8B-Instruct^30^, Starling-7B-alpha^31^, Starling-7B-beta^32^), including two further trained on biomedical text (BioMistral-7B^33^, JSL-MedMNX-7B-SFT^34^). Of these models, Gemma2-9B-it showed the best performance with the highest microF1 scores for both medication start (0.806) and stop (0.882) extraction (Supplementary Table 8).

### GPT-4 contraceptive switching information extraction outperforms baseline models

**Fig. 3:**
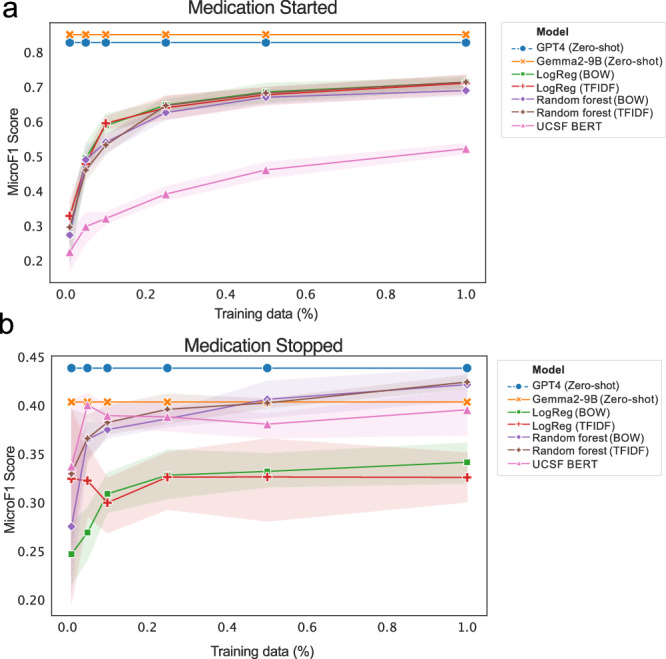
GPT-4 performance compared to baseline. Following prompt evaluation, GPT-4 performance on the remaining test set was also compared to baseline model performance for extraction of contraceptive (**a**) started and **b** stopped. Silver-standard labels from structured data were used for training and evaluation of baseline models, and for evaluation of zero-shot GPT-4.

Zero-shot GPT-4 performance using the best prompt was also compared to baseline models trained on different proportions silver-standard labels derived from structured data. GPT-4 outperformed all baseline models, regardless of the proportion of training data used for baseline models (Fig. 3), with micro F1 scores of 0.828 and 0.439 on contraceptive start and stop extraction, respectively. The next best model was random forest trained on TF-IDF representations, with a 0.714 (SD: 0.024) score on medication start and 0.424 (SD: 0.009) on medication stopping. The cost for running all GPT-4 values, including prompt development and inference for the test set was $78.40 based on a cost of $0.03 per 1000 input tokens and $0.06 per 1000 output tokens.

Concordance between silver-standard labels and human annotations available showed a Cohen’s Kappa coefficient of 0.585 for medication starting labels and 0.217 for contraceptive stopping (*n* = 93). When we removed notes without relevant contraceptives, determined by the human evaluator, concordance between these two methods increased to 0.960 for contraceptives started and 0.644 for contraceptives stopped (Supplementary Table 5).

### Identification of reasons for contraceptive switching

**Fig. 4:**
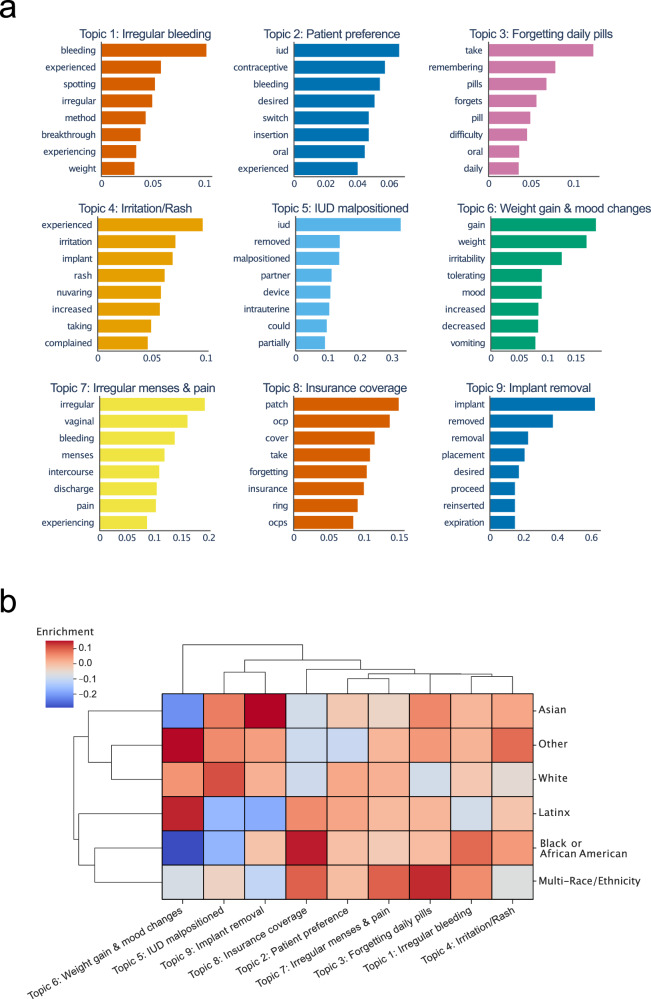
Clustering reasons for contraceptive switching using BERTopic. **a** BERTopic modeling was used to cluster GPT-4 extracted reasons for contraceptive switching, with nine key topics identified. Top terms for each cluster are shown. **b** Topics were assessed for enrichment amongst patient subgroups by race/ethnicity. Higher enrichment scores indicate higher prevalence of a topic written in notes within a patient subgroup.

Unsupervised BERTopic topic modeling of extracted reasons for stopping across the full dataset identified 19 topics, which were manually grouped into 10 cohesive topics (Supplementary Table 6). Excluding the 1136 notes that did not contain a relevant reason (topic 0, Supplementary Table 7), the most frequently occurring topics contained terms related to spotting and irregular bleeding (topic 1, *n* = 272), desire to switch contraceptives (topic 2), and forgetting to take daily pills (topic 3, *n* = 272). Topics 4 (*n* = 68), 6 (*n* = 21), and 7 (*n* = 21) described other adverse events of contraceptive use, including irritation and rash, weight gain and mood changes, and irregular menses and pain. Topic 5 (*n* = 31) related to IUD malpositioning and removal, and topic 9 (*n* = 12) related to implant removal. Finally, topic 8 included terms related to insurance coverage (Fig. 4a).

Topic clusters of reasons for switching were analyzed for enrichment in patient subsets stratified by race/ethnicity and age, both of which have been shown to be associated with differences in contraceptive selection^2,5,35^. Weight gain and mood change (topic 6) were enriched in patients who self-reported as being “Latinx” or “Other”, and were less prevalent in patients self-reporting as “Black or African American”. Topic 9 (Implant removal) was enriched in patients who self-reported a race/ethnicity of “Asian”, and topic 8 (insurance coverage) was enriched in patients of “Black or African American”, “Latinx”, or “Multi-Race/Ethnicity” race/ethnicity (Fig. 4b). When stratified by age, we found that patients in the “<21” age group tended to show enrichment in topic 8 (Insurance coverage) while patients in the “40 + ” age group were more likely to switch based on reasons in topic 6 (Weight gain and mood changes, Supplementary Fig. 1).

## Discussion

We demonstrated that large language models can accurately extract treatment switching rationales from associated clinical notes with clear implications for better understanding of patient care. In the task of treatment switches, GPT-4 performance, evaluated by both gold-standard manual annotation and automated analysis, was stable between six different prompts. We further showed that the vast majority of reasons for contraceptive switching extracted by GPT-4 were correct, with minimal hallucinations. Finally, we uncovered latent contraceptive-specific reasons for switching medications by clustering embeddings derived from GPT-4 extracted values.

While switching contraceptives is not inherently negative, understanding the rationales behind these clinical decisions is crucial to closing potential gaps in care. Topic clusters ranged from treatment failure to patient preference, as well as adverse events and insurance reasons. We showed that insurance coverage as a reason for switching disproportionately affected patients identifying as “Latinx” or “Black or African American” while switching due to IUD misplacement was enriched in patients identifying as “White” or “Asian”. Additionally, we showed that weight gain and mood changes as reasons for switching were enriched in patient populations who self-reported their race/ethnicity as “Latinx” or “Other”. A previous study of weight gain with progestin-only contraceptive use also found that only patients who reported their race as “Black” showed statistically significant weight gain over 12 months^36^, but did not provide “Latinx” as a category for race and did not look at discontinuation rates. These and our findings prompt the need for further investigation of weight gain with contraceptive use in diverse populations to better understand the factors driving contraceptive usage and switching. Other future work could investigate similar patterns in other conditions and medication treatment protocols, as well as how switching may differ in patients stratified by other factors, such as income or insurance status, which were not considered here due to sparsity in the dataset.

The implications of our work on clinical research and practice are threefold. First, our findings can increase clinicians’ awareness of the diverse reasons for treatment switching, ranging from patient preferences and adverse events to insurance coverage issues. While the extracted rationales are not necessarily causal, this enhanced understanding may help healthcare providers better anticipate potential challenges with contraceptive usage. Second, by recognizing these factors in advance, clinicians may be able to improve their initial treatment selection process, potentially reducing the frequency of switches and enhancing patient satisfaction. Lastly, this knowledge can be used to set more accurate expectations for patients. By incorporating these insights into clinical practice, healthcare providers can optimize contraceptive management, leading to improved patient outcomes and experiences.

While our study results highlight recent medical concerns regarding financial barriers to contraceptive access and socioeconomic inequities in reproductive health^5,8,37^, there are several limitations to consider. Our dataset is limited by sample size for contraceptive switching prediction and is derived from a large, academic medical center, which may introduce bias in the types of patients or contraceptives captured. We also assume that clinical notes contain information on all medications ordered at the same or previous encounters, but some medications may not be discussed or documented. This is reflected in poor concordance between structured data labels and human evaluation, particularly for medication stopping values. Some contraceptives are also intended for longer-term use, which may affect time to switching. Additionally, because the de-identification process is not perfect, manual review of some notes identified several medication names that were inappropriately redacted. This was particularly prevalent among contraceptive brand names that resemble common patient names (eg. “Camila”^38^ or “Heather”^39^) that are deliberately redacted. Another limitation is that the medical history of each patient is not static per patient, and confounding diagnoses or other relevant medical history were not considered in this analysis. This study also grouped together contraceptives by modality and analyzing switching within modalities could provide ample grounds for future work. Additionally, while this study surfaces associations between specific demographic subpopulations and contraceptive switching reasons, causal analyses and interventional approaches to address such disparities will require further study.

Finally, although LLMs demonstrate great promise and performance on many key clinical tasks^21,40,41^, another key limitation of our work surrounds the nature of LLMs, which can lack transparency about training data, model development, and evaluation. There is little public information provided about GPT4’s training data, approach, or model architecture. As a result, we refrain from making conclusions about why LLMs like the GPT-4 model produce certain results, and focus instead on evaluating overall performance and insights that can be derived from extraction of information from clinical notes. Additionally, although LLMs offer human-like input and output, the decision making processes of the models lack meaningful interpretability, which is of significant importance to clinical care. Our work’s impact on clinical practice and public health should be considered through the lens of these limitations and concerns.

In conclusion, this study reveals differences in the reasons behind contraceptive switching using information extracted from clinical notes with a large language model. We showed that specific underserved demographic groups are more likely to switch due to issues like insurance coverage limitations or adverse events, going beyond information only captured in structured medical record data. While our understanding of reasons for contraceptive switching will require external validation, the computational approach developed here enables a data-driven understanding of what drives treatment decisions and where disparities may exist. More broadly, these methods can unlock patient perspectives and values, moving towards more patient-centered care. As we apply larger and more complex models to healthcare data, we must intentionally use these methods to better understand heterogeneous patient populations.

## Methods

### Contraceptive switching cohort selection

A contraceptive switching cohort was selected from the UCSF Information Commons dataset, which contains deidentified structured data and clinical notes from over 6 million patients between 2012–2023. Clinical text notes were certified as deidentified as previously described^25^ and are usable by UCSF researchers as non-human subjects research, determined to be exempt from further review.

We identified all patients prescribed at least one contraceptive documented in the structured medication data based on a “therapeutic class” label. Non-drug contraceptives (e.g diaphragms/cervical caps, condoms, vaginal pH modulators, and spermicides), progestin and estrogen-containing agents not used for contraceptive purposes, and emergency contraceptives were removed (Supplementary Table 1). The remaining contraceptives were mapped to the following modalities: Oral, Implant, Intrauterine device (IUD), Injection (intramuscular or subcutaneous), Transdermal, and Intravaginal based on regular expression values (Supplementary Table 2). Contraceptives prescribed without a start date or associated clinical note and duplicate orders at each encounter date were removed. To filter out short notes without any relevant information, only clinical notes containing >50 tokens, created using encodings from OpenAI’s open-source tokenizer tiktoken^42^.

The dataset was further filtered to patients with encounters at least 6 months after the prescription of the first contraceptive, ensuring those without a switch weren’t lost to follow-up. Prescriptions were sorted by documented start date, and encounters that contained a contraceptive switch were retrieved. A contraceptive switch was defined as a difference in prescribed contraceptive modalities between consecutive encounters.

Self-reported demographic information on race/ethnicity and preferred language were extracted from structured data, which was also used to calculate age at date of first contraceptive prescription. This study was conducted using retrospective, deidentified clinical data and was determined to be exempt from IRB review. All data were stored or processed on HIPAA compliant hardware at UCSF or through a HIPAA compliant Microsoft Azure instance (“UCSF Versa”). No data was transferred or stored by OpenAI; and OpenAI settings were maintained so that no prompt information would be stored, even temporarily.

### Prompt evaluation for extraction of contraceptive selection strategy

Prompting can have significant effects on the accuracy of large language models^43,44^. We tested six prompts (Supplementary Table 3), varying both system information and output formats, to extract the following information: (1) which contraceptive was stopped, (2) which new contraceptive was started, and (3) why the contraceptive switch occurred. To avoid overfitting, these six prompts were evaluated on a held-out subset of contraceptive switching clinical notes from 5% of the patients. The model used was GPT-4, with temperature set at 0, maximum response length capped at 500 tokens, top_p set to 1, and all other parameters kept as default. A zero-shot approach was used, with no additional information or training data provided outside of the encounter’s associated clinical note. Resulting values were mapped to the six contraceptive modalities using regular expression values (Supplementary Table 2). All GPT-4 queries were performed using the “0613” version of GPT-4 and were run between November 13–15, 2023.

A clinical evaluator (EE) assessed the accuracy of GPT-4 extraction for contraceptives started and stopped within each note. Micro F1 scores, which represent the harmonic mean of precision and recall scores, are reported. The best prompt was selected based on the highest average score attained across all medications started/stopped determined by manual evaluation. This prompt was also used to test the performance of several open-source language models^26–34^. Greedy sampling, 8-bit quantization, and a maximum response length of 250 was used for all open-source models.

For evaluation extracted reasons for GPT-4, the clinical reviewer was also instructed to identify whether the extracted reason was accurate based on the clinical note and whether any hallucination occurred, which was defined as information produced by the language model that could not be derived from the clinical note.

### Comparison of GPT-4 contraceptive information extraction to baseline models

The best prompt selected from the development dataset was applied to the remaining 95% “test set” of the contraceptive switching cohort using the same GPT-4 setup. We compared our LLM-based methods against several traditional machine learning techniques, including logistic regression, random forest, and BERT-style models. Since human clinical annotations were not available for this larger dataset, weak labels from structured data, specifically which contraceptives were started and stopped at the associated clinical encounter, were used for training and evaluation in each of these models. Structured data may not reflect the contents of clinical notes if patients are prescribed contraceptives at a different facility or stop dates are not documented, so we compared these silver-standard labels to human annotation for the 93 clinical notes in the prompt evaluation set using Cohen’s Kappa coefficient to assess reliability between the two sources.

Two sets of logistic regression and random forest models were developed using either bag-of-words and term-frequency inverse document frequency (TF-IDF)^45^ text representations. Multiclass classification was performed, with models predicting the modality of contraceptives started or stopped (oral, IUD, subdermal, intravaginal, injection, transdermal). We performed 5-fold cross validation using a 70/10/20 split between train, validation, and test data. Due to differences in training sizes between baseline models and GPT-4, this split is independent of the previous prompt evaluation and GPT-4 test sets. Hyperparameter tuning was performed using a grid search of varying regularization values (C = [0.01, 0.1, 1, 10, 100, 1000]) for logistic regression and both number of estimators and max depth for random forest (n_estimators = [50, 100, 250, 500], max_depth = [20, 50, 100]).

The UCSF-BERT model^46^ trained on a large corpus of clinical notes was also used as a baseline. Again, we performed 5-fold cross validation using a 70/10/20 split. Hyperparameter tuning was performed using Optuna^47^, and both learning rate and weight decay were varied (learning rate = (1e-5, 5e-5), weight decay = (4e-5, 0.01)). Models were trained for 5 epochs, with early stopping. To accommodate for the 512 maximum token length allowed by UCSF BERT, a sliding window was used with final prediction selected by majority vote across all windows.

To simulate few-shot learning, we trained each of the baseline models on random subsamples of 100%, 50%, 25%, 10%, 5%, and 1% of the training data. Micro-averaged F1 scores are reported for each model on the held-out test set.

### Unsupervised clustering of extracted reasons for contraceptive switching

GPT-4 was also used to extract reasons for contraceptive switching from the test set using the best prompt. To identify key reasons for medication switching, we applied BERTopic, a topic modeling method that clusters document embeddings, to all reasons extracted from both the prompt evaluation and test sets. The UCSF-BERT model was used to generate embeddings from the list of extracted reasons and embeddings were clustered by BERTopic^45^. Briefly, dimensionality reduction was applied to the embeddings using Uniform Manifold Approximation and Projection^48^ (UMAP), with 5 components and 3 neighbors with euclidean distance metrics. HDBSCAN^49^ was used to cluster reduced embeddings, with the number of topics dynamically chosen by the algorithm using “auto” settings for nr_topics parameter, and TF-IDF used to identify key terms from each cluster. All other default parameters were used. Topics were manually reviewed and similar topics based on the top 10 most frequent terms in each topic were grouped together.

Subgroup analysis was performed to understand whether topics were associated with specific patient demographics. Adapting from previous enrichment methods^50^, we used topic probabilities assigned to each document by the BERTopic model to calculate a weighted enrichment score that describes the relative contribution of each topic to patient subsets. Specifically, enrichment scores were calculated as ${\theta }_{k,j}=\frac{{q}_{n,k}\,\cdot \,{y}_{n,j}}{{\sum }_{n=1}^{N}{q}_{n,k}\,* \,{\sum }_{n=1}^{N}{y}_{n,j}}$, where q(n,k) describes the weight of each topic k for note n, and y(n,j) are the patient subsets assigned to each note. The scores were normalized by total topic weight, as well as by number of patients in each subset, and reported scores were log transformed. The same analysis was performed for patients stratified by age group, which were split into categories “<21”, “21–30”, “31–40”, and “40 + ”.

### Statistics

We present means and standard deviations for continuous distribution, and utilize two-sided *t*-tests to analyze differences in continuous distributions. To evaluate differences in categorical data, Chi-square tests were applied. Statistical analyses were conducted using the SciPy package^51^, and a *p*-value <0.05 was used to indicate statistical significance.

## Supplementary information

Supplementary Material

## Data Availability

Clinical notes from this study are not publicly available, except for a subset of GPT4 extracted reasons for contraceptive switching from clinical notes.

## Code availability

All code to reproduce the methods described here are made available at https://github.com/BMiao10/contraceptive-switching.
