# Supplementary material for: Understanding contraceptive switching rationales from real world clinical notes using large language models
Source: NPJ Digit Med. 2025 Apr 23;8:221. doi: 10.1038/s41746-025-01615-0 (PMC12019358; doi:10.1038/s41746-025-01615-0)
Supplement: Supplementary file 1 — Supplementary Material [file 41746_2025_1615_MOESM1_ESM.pdf]

Supplementary Information

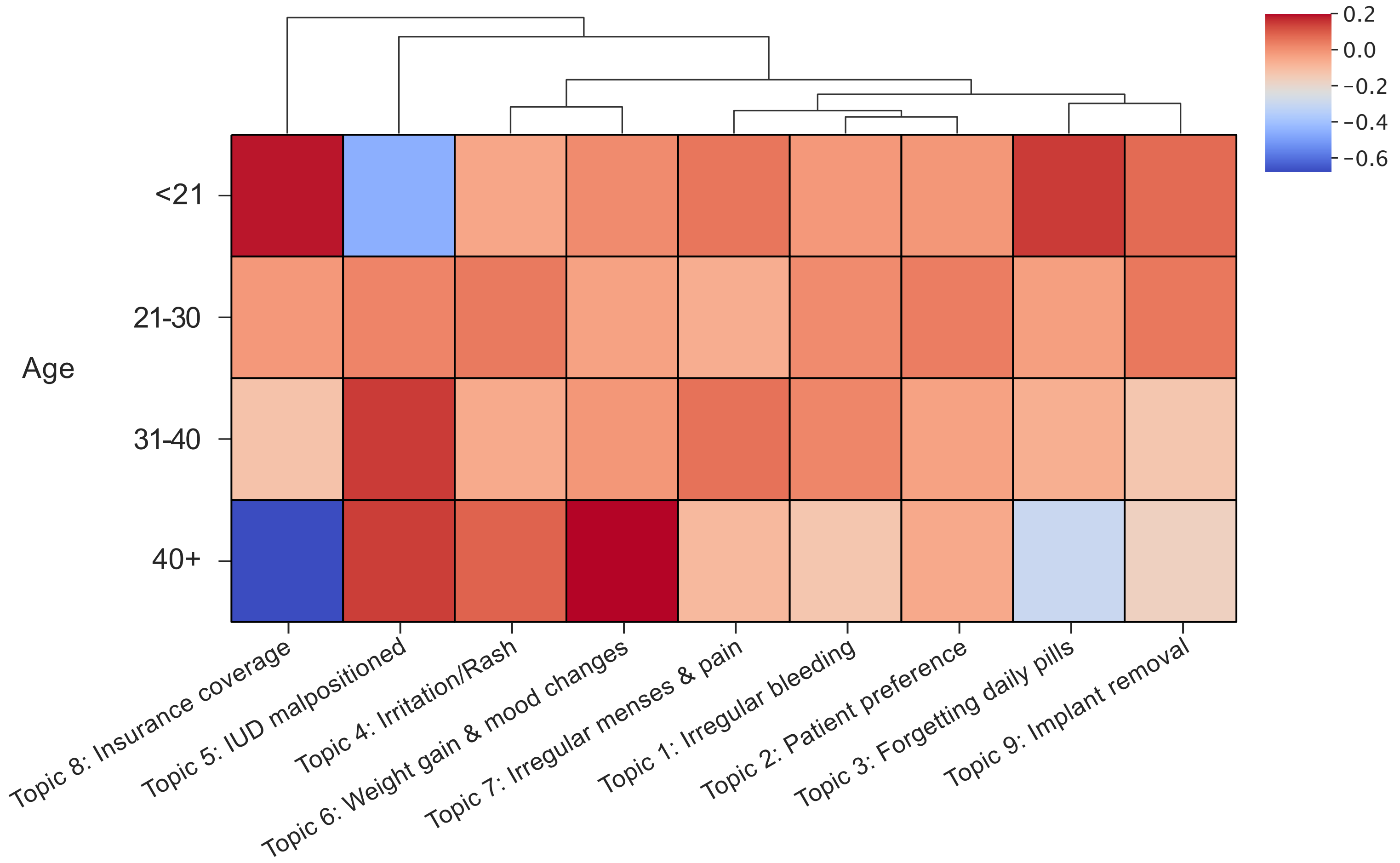

Supplementary Figure 1: Enrichment of reasons for contraceptive switching in patients in varying age groups.
Heatmap showing enrichment scores for reasons of contraceptive switching across different age categories. Topics were identified using BERTopic, a document embedding clustering algorithm. Enrichment scores describe the relative contribution of each topic within patient subsets, normalized by topic weight and patient group size. Topic 8 (Insurance coverage) was enriched in younger patients (<21) while weight gain and mood changes (topic 6) as a reason for switching was more common in older patients (40+).

Supplementary Table 1: Contraceptive medication pharmaceutical classes removed.
Contraceptive medication classes that were removed for the patient cohort considered. These classes included non-pharmaceutical agents (e.g diaphragms/cervical caps, condoms, vaginal pH modulators, and spermicides), progestin and estrogen-containing agents not used for contraceptive purposes or with no clear modality, and emergency contraceptives.

Medication name	Medication Pharmaceutical Class	Medication Pharmaceutical Subclass
AIMSCO LATEX CONDOM	CONDOMS	Medical Supplies and DME - Male Condoms
AIMSCO MISC	CONDOMS	Medical Supplies and DME - Male Condoms
CONDOMS - FEMALE	CONDOMS	Medical Supplies and DME - Female Condoms
CONDOMS - FEMALE MISC	CONDOMS	Medical Supplies and DME - Female Condoms
CONDOMS - MALE	CONDOMS	Medical Supplies and DME - Male Condoms
CONDOMS - MALE MISC	CONDOMS	Medical Supplies and DME - Male Condoms
CONDOMS LATEX LUBRICATED	CONDOMS	Medical Supplies and DME - Male Condoms
CONDOMS LATEX NON-LUBRICATED	CONDOMS	Medical Supplies and DME - Male Condoms
CONDOMS NON-LATEX NON-LUBRICAT	CONDOMS	Medical Supplies and DME - Male Condoms
CONDOMS, FEMALE	CONDOMS	Medical Supplies and DME - Female Condoms
CONDOMS, NON-LATEX, LUBRICATED	CONDOMS	Medical Supplies and DME - Male Condoms
CONDOMS-PREM LUBRICATED	CONDOMS	Medical Supplies and DME - Male Condoms
DUREX AVANTI BARE REAL FEEL CONDOM	CONDOMS	Medical Supplies and DME - Male Condoms
FC2 FEMALE CONDOM	CONDOMS	Medical Supplies and DME - Female Condoms
FC2 FEMALE CONDOM MISC	CONDOMS	Medical Supplies and DME - Female Condoms
KIMONO MAXX CONDOMS	CONDOMS	Medical Supplies and DME - Male Condoms
TRUSTEX-RIA LUB/SPERMICIDE MISC	CONDOMS	Medical Supplies and DME - Male Condoms
CAYA CONTOURED 65 MM-80 MM VAGINAL DIAPHRAGM	DIAPHRAGMS/CERVICAL CAP	Medical Supplies and DME - Diaphragms
CERVICAL CAPS 22 MM	DIAPHRAGMS/CERVICAL CAP	Medical Supplies and DME - Cervical Caps
CERVICAL CAPS 25 MM	DIAPHRAGMS/CERVICAL CAP	Medical Supplies and DME - Cervical Caps
CERVICAL CAPS 26 MM	DIAPHRAGMS/CERVICAL CAP	Medical Supplies and DME - Cervical Caps
CERVICAL CAPS 30 MM	DIAPHRAGMS/CERVICAL CAP	Medical Supplies and DME - Cervical Caps
DIAPHRAGM ARC-SPRING 65 MM KIT	DIAPHRAGMS/CERVICAL CAP	Medical Supplies and DME - Diaphragms
DIAPHRAGM ARC-SPRING 70 MM KIT	DIAPHRAGMS/CERVICAL CAP	Medical Supplies and DME - Diaphragms
DIAPHRAGM ARC-SPRING 70 MM VAGINAL	DIAPHRAGMS/CERVICAL CAP	Medical Supplies and DME - Diaphragms
DIAPHRAGM ARC-SPRING 75 MM KIT	DIAPHRAGMS/CERVICAL CAP	Medical Supplies and DME - Diaphragms
DIAPHRAGM ARC-SPRING 80 MM KIT	DIAPHRAGMS/CERVICAL CAP	Medical Supplies and DME - Diaphragms
DIAPHRAGM ARC-SPRING VAGINAL	DIAPHRAGMS/CERVICAL CAP	Medical Supplies and DME - Diaphragms
DIAPHRAGM COIL SPRING VAGINAL	DIAPHRAGMS/CERVICAL CAP	Medical Supplies and DME - Diaphragms
DIAPHRAGM FITTING SET, ARC-SPRING VAGINAL	DIAPHRAGMS/CERVICAL CAP	Medical Supplies and DME - Diaphragms
DIAPHRAGM WIDE SEAL 65 MM VAGINAL	DIAPHRAGMS/CERVICAL CAP	Medical Supplies and DME - Diaphragms
DIAPHRAGM WIDE SEAL 70 MM VAGINAL	DIAPHRAGMS/CERVICAL CAP	Medical Supplies and DME - Diaphragms
DIAPHRAGM WIDE SEAL 75 MM VAGINAL	DIAPHRAGMS/CERVICAL CAP	Medical Supplies and DME - Diaphragms
DIAPHRAGM WIDE SEAL 80 MM VAGINAL	DIAPHRAGMS/CERVICAL CAP	Medical Supplies and DME - Diaphragms
DIAPHRAGMS, CONTOURED 65 MM-80 MM VAGINAL	DIAPHRAGMS/CERVICAL CAP	Medical Supplies and DME - Diaphragms
FEMCAP 26 MM VAGINAL DEVICE	DIAPHRAGMS/CERVICAL CAP	Medical Supplies and DME - Cervical Caps
ORTHO ALL-FLEX FITTING SET VAGINAL	DIAPHRAGMS/CERVICAL CAP	Medical Supplies and DME - Diaphragms
ORTHO DIAPHRAGM ALL-FLEX 65 65 MM KIT	DIAPHRAGMS/CERVICAL CAP	Medical Supplies and DME - Diaphragms
ORTHO DIAPHRAGM ALL-FLEX 70 70 MM KIT	DIAPHRAGMS/CERVICAL CAP	Medical Supplies and DME - Diaphragms
ORTHO DIAPHRAGM ALL-FLEX 75 VAGINAL	DIAPHRAGMS/CERVICAL CAP	Medical Supplies and DME - Diaphragms
WIDE-SEAL DIAPHRAGM 75 MM VAGINAL	DIAPHRAGMS/CERVICAL CAP	Medical Supplies and DME - Diaphragms
ACTIVELLA 0.5 MG-0.1 MG TABLET	ESTROGENIC AGENTS	Estrogen-Progestin
ACTIVELLA 1 MG-0.5 MG TABLET	ESTROGENIC AGENTS	Estrogen-Progestin
ACTIVELLA ORAL	ESTROGENIC AGENTS	Estrogen-Progestin
ALORA 0.025 MG/24 HR TRANSDERMAL PATCH	ESTROGENIC AGENTS	Estrogens
ALORA 0.05 MG/24 HR TRANSDERMAL PATCH	ESTROGENIC AGENTS	Estrogens
ALORA 0.075 MG/24 HR TRANSDERMAL PATCH	ESTROGENIC AGENTS	Estrogens
ALORA 0.1 MG/24 HR TRANSDERMAL PATCH	ESTROGENIC AGENTS	Estrogens
ALORA TD	ESTROGENIC AGENTS	Estrogens
AMABELZ 0.5 MG-0.1 MG TABLET	ESTROGENIC AGENTS	Estrogen-Progestin
AMABELZ 1 MG-0.5 MG TABLET	ESTROGENIC AGENTS	Estrogen-Progestin
AMABELZ ORAL	ESTROGENIC AGENTS	Estrogen-Progestin
CENESTIN 0.3 MG TABLET	ESTROGENIC AGENTS	Estrogens
CENESTIN 0.625 MG TABLET	ESTROGENIC AGENTS	Estrogens
CENESTIN ORAL	ESTROGENIC AGENTS	Estrogens
CLIMARA 0.025 MG/24 HR TRANSDERMAL PATCH	ESTROGENIC AGENTS	Estrogens
CLIMARA 0.0375 MG/24 HR TRANSDERMAL PATCH	ESTROGENIC AGENTS	Estrogens

CLIMARA 0.05 MG/24 HR TRANSDERMAL PATCH	ESTROGENIC AGENTS	Estrogens
CLIMARA 0.06 MG/24 HR TRANSDERMAL PATCH	ESTROGENIC AGENTS	Estrogens
CLIMARA 0.075 MG/24 HR TRANSDERMAL PATCH	ESTROGENIC AGENTS	Estrogens
CLIMARA 0.1 MG/24 HR TRANSDERMAL PATCH	ESTROGENIC AGENTS	Estrogens
CLIMARA PRO 0.045 MG-0.015 MG/24 HR TRANSDERMAL PATCH	ESTROGENIC AGENTS	Estrogen-Progestin
CLIMARA PRO TD	ESTROGENIC AGENTS	Estrogen-Progestin
CLIMARA TD	ESTROGENIC AGENTS	Estrogens
COMBIPATCH 0.05 MG-0.14 MG/24 HR TRANSDERMAL	ESTROGENIC AGENTS	Estrogen-Progestin
COMBIPATCH 0.05 MG-0.25 MG/24 HR TRANSDERMAL	ESTROGENIC AGENTS	Estrogen-Progestin
COMBIPATCH TD	ESTROGENIC AGENTS	Estrogen-Progestin
CONJ ESTROGEN-MEDROXYPROGESTERONE 0.3 MG-1.5 MG TABLET	ESTROGENIC AGENTS	Estrogen-Progestin
CONJ ESTROGEN-MEDROXYPROGESTERONE 0.45 MG-1.5 MG TABLET	ESTROGENIC AGENTS	Estrogen-Progestin
CONJ ESTROGEN-MEDROXYPROGESTERONE 0.625 MG(14)/0.625 MG-5MG(14) TABLET	ESTROGENIC AGENTS	Estrogen-Progestin
CONJ ESTROGEN-MEDROXYPROGESTERONE 0.625 MG-2.5 MG TABLET	ESTROGENIC AGENTS	Estrogen-Progestin
CONJ ESTROGEN-MEDROXYPROGESTERONE 0.625 MG-5 MG TABLET	ESTROGENIC AGENTS	Estrogen-Progestin
CONJUGATED ESTROGENS 0.3 MG TABLET	ESTROGENIC AGENTS	Estrogens
CONJUGATED ESTROGENS 0.45 MG TABLET	ESTROGENIC AGENTS	Estrogens
CONJUGATED ESTROGENS 0.625 MG TABLET	ESTROGENIC AGENTS	Estrogens
CONJUGATED ESTROGENS 0.9 MG TABLET	ESTROGENIC AGENTS	Estrogens
CONJUGATED ESTROGENS 1.25 MG TABLET	ESTROGENIC AGENTS	Estrogens
CONJUGATED ESTROGENS 2.5 MG TABLET	ESTROGENIC AGENTS	Estrogens
CONJUGATED ESTROGENS 25 MG SOLUTION FOR INJECTION	ESTROGENIC AGENTS	Estrogens
CONJUGATED ESTROGENS INJ	ESTROGENIC AGENTS	Estrogens
CONJUGATED ESTROGENS ORAL	ESTROGENIC AGENTS	Estrogens
DELESTROGEN 10 MG/ML INTRAMUSCULAR OIL	ESTROGENIC AGENTS	Estrogens
DELESTROGEN 20 MG/ML INTRAMUSCULAR OIL	ESTROGENIC AGENTS	Estrogens
DELESTROGEN 40 MG/ML INTRAMUSCULAR OIL	ESTROGENIC AGENTS	Estrogens
DELESTROGEN IM	ESTROGENIC AGENTS	Estrogens
DEPO-ESTRADIOL 5 MG/ML INTRAMUSCULAR OIL	ESTROGENIC AGENTS	Estrogens
DEPO-ESTRADIOL IM	ESTROGENIC AGENTS	Estrogens
DIVIGEL 0.25 MG/0.25 GRAM (0.1 %) TRANSDERMAL GEL PACKET	ESTROGENIC AGENTS	Estrogens
DIVIGEL 0.5 MG/0.5 GRAM (0.1 %) TRANSDERMAL GEL PACKET	ESTROGENIC AGENTS	Estrogens
DIVIGEL 0.75 MG/0.75 GRAM (0.1%) TRANSDERMAL GEL PACKET	ESTROGENIC AGENTS	Estrogens
DIVIGEL 1 MG/GRAM (0.1 %) TRANSDERMAL GEL PACKET	ESTROGENIC AGENTS	Estrogens
DIVIGEL TD	ESTROGENIC AGENTS	Estrogens
DOTTI 0.025 MG/24 HR TRANSDERMAL PATCH	ESTROGENIC AGENTS	Estrogens
DOTTI 0.0375 MG/24 HR TRANSDERMAL PATCH	ESTROGENIC AGENTS	Estrogens
DOTTI 0.05 MG/24 HR TRANSDERMAL PATCH	ESTROGENIC AGENTS	Estrogens
DOTTI 0.075 MG/24 HR TRANSDERMAL PATCH	ESTROGENIC AGENTS	Estrogens
DOTTI 0.1 MG/24 HR TRANSDERMAL PATCH	ESTROGENIC AGENTS	Estrogens
DOTTI TD	ESTROGENIC AGENTS	Estrogens
ELESTRIN 0.87 GRAM/ACTUATION (0.06%) TRANSDERMAL GEL PUMP	ESTROGENIC AGENTS	Estrogens
ELESTRIN TD	ESTROGENIC AGENTS	Estrogens
ENJUVIA 0.3 MG TABLET	ESTROGENIC AGENTS	Estrogens
ENJUVIA 0.625 MG TABLET	ESTROGENIC AGENTS	Estrogens
ENJUVIA 0.9 MG TABLET	ESTROGENIC AGENTS	Estrogens
ENJUVIA 1.25 MG TABLET	ESTROGENIC AGENTS	Estrogens
ENJUVIA ORAL	ESTROGENIC AGENTS	Estrogens
ESTERIFIED ESTROGENS 0.3 MG TABLET	ESTROGENIC AGENTS	Estrogens
ESTERIFIED ESTROGENS 0.625 MG TABLET	ESTROGENIC AGENTS	Estrogens
ESTERIFIED ESTROGENS 1.25 MG TABLET	ESTROGENIC AGENTS	Estrogens
ESTERIFIED ESTROGENS 2.5 MG TABLET	ESTROGENIC AGENTS	Estrogens
ESTERIFIED ESTROGENS ORAL	ESTROGENIC AGENTS	Estrogens
ESTRACE 0.5 MG TABLET	ESTROGENIC AGENTS	Estrogens

ESTRACE 1 MG TABLET	ESTROGENIC AGENTS	Estrogens
ESTRACE 2 MG TABLET	ESTROGENIC AGENTS	Estrogens
ESTRACE ORAL	ESTROGENIC AGENTS	Estrogens
ESTRADERM 0.1 MG/24 HR TRANSDERMAL PATCH	ESTROGENIC AGENTS	Estrogens
ESTRADERM TD	ESTROGENIC AGENTS	Estrogens
ESTRADIOL 0.025 MG/24 HR SEMIWEEKLY TRANSDERMAL PATCH	ESTROGENIC AGENTS	Estrogens
ESTRADIOL 0.025 MG/24 HR WEEKLY TRANSDERMAL PATCH	ESTROGENIC AGENTS	Estrogens
ESTRADIOL 0.0375 MG/24 HR SEMIWEEKLY TRANSDERMAL PATCH	ESTROGENIC AGENTS	Estrogens
ESTRADIOL 0.0375 MG/24 HR WEEKLY TRANSDERMAL PATCH	ESTROGENIC AGENTS	Estrogens
ESTRADIOL 0.045 MG-LEVONORGESTREL 0.015 MG/24HR WEEKLY TRANSDERM PATCH	ESTROGENIC AGENTS	Estrogen-Progestin
ESTRADIOL 0.05 MG-NORETHINDRONE 0.14 MG/24 HR SEMIWKLY TRANSDERM PATCH	ESTROGENIC AGENTS	Estrogen-Progestin
ESTRADIOL 0.05 MG-NORETHINDRONE 0.25 MG/24 HR SEMIWKLY TRANSDERM PATCH	ESTROGENIC AGENTS	Estrogen-Progestin
ESTRADIOL 0.05 MG/24 HR SEMIWEEKLY TRANSDERMAL PATCH	ESTROGENIC AGENTS	Estrogens
ESTRADIOL 0.05 MG/24 HR WEEKLY TRANSDERMAL PATCH	ESTROGENIC AGENTS	Estrogens
ESTRADIOL 0.06 MG/24 HR WEEKLY TRANSDERMAL PATCH	ESTROGENIC AGENTS	Estrogens
ESTRADIOL 0.075 MG/24 HR SEMIWEEKLY TRANSDERMAL PATCH	ESTROGENIC AGENTS	Estrogens
ESTRADIOL 0.075 MG/24 HR WEEKLY TRANSDERMAL PATCH	ESTROGENIC AGENTS	Estrogens
ESTRADIOL 0.1 MG/24 HR SEMIWEEKLY TRANSDERMAL PATCH	ESTROGENIC AGENTS	Estrogens
ESTRADIOL 0.1 MG/24 HR WEEKLY TRANSDERMAL PATCH	ESTROGENIC AGENTS	Estrogens
ESTRADIOL 0.25 MG/0.25 GRAM (0.1 %) TRANSDERMAL GEL PACKET	ESTROGENIC AGENTS	Estrogens
ESTRADIOL 0.5 MG TABLET	ESTROGENIC AGENTS	Estrogens
ESTRADIOL 0.5 MG/0.5 GRAM (0.1 %) TRANSDERMAL GEL PACKET	ESTROGENIC AGENTS	Estrogens
ESTRADIOL 0.75 MG/0.75 GRAM (0.1%) TRANSDERMAL GEL PACKET	ESTROGENIC AGENTS	Estrogens
ESTRADIOL 0.87 GRAM/ACTUATION (0.06%) TRANSDERMAL GEL PUMP	ESTROGENIC AGENTS	Estrogens
ESTRADIOL 1 MG TABLET	ESTROGENIC AGENTS	Estrogens
ESTRADIOL 1 MG/GRAM (0.1 %) TRANSDERMAL GEL PACKET	ESTROGENIC AGENTS	Estrogens
ESTRADIOL 1.25 GRAM/ACTUATION (0.06%) TRANSDERMAL GEL PUMP	ESTROGENIC AGENTS	Estrogens
ESTRADIOL 1.25 MG/1.25 GRAM (0.1 %) TRANSDERMAL GEL PACKET	ESTROGENIC AGENTS	Estrogens
ESTRADIOL 1.5 MG TABLET	ESTROGENIC AGENTS	Estrogens
ESTRADIOL 1.53 MG/SPRAY (1.7 %) TRANSDERMAL SPRAY	ESTROGENIC AGENTS	Estrogens
ESTRADIOL 10 MG IMPLANT PELLET	ESTROGENIC AGENTS	Estrogens
ESTRADIOL 14 MCG/24 HR WEEKLY TRANSDERMAL PATCH	ESTROGENIC AGENTS	Estrogens
ESTRADIOL 2 MG TABLET	ESTROGENIC AGENTS	Estrogens
ESTRADIOL 2.5 MG/GRAM (1.74 GRAM) TRANSDERMAL EMULSION PACKET	ESTROGENIC AGENTS	Estrogens
ESTRADIOL ACETATE 0.45 MG TABLET	ESTROGENIC AGENTS	Estrogens
ESTRADIOL ACETATE 0.9 MG TABLET	ESTROGENIC AGENTS	Estrogens
ESTRADIOL ACETATE ORAL	ESTROGENIC AGENTS	Estrogens
ESTRADIOL CYPIONATE 5 MG/ML INTRAMUSCULAR OIL	ESTROGENIC AGENTS	Estrogens
ESTRADIOL CYPIONATE IM	ESTROGENIC AGENTS	Estrogens
ESTRADIOL IMPLANT	ESTROGENIC AGENTS	Estrogens
ESTRADIOL ORAL	ESTROGENIC AGENTS	Estrogens
ESTRADIOL TD	ESTROGENIC AGENTS	Estrogens
ESTRADIOL TRANSDERMAL PATCH 0.05 MG/24 HR	ESTROGENIC AGENTS	Estrogens
ESTRADIOL TRANSDERMAL PATCH 0.1 MG/24 HR	ESTROGENIC AGENTS	Estrogens
ESTRADIOL TRANSDERMAL PATCH TD	ESTROGENIC AGENTS	Estrogens
ESTRADIOL VALERATE 10 MG/ML INTRAMUSCULAR OIL	ESTROGENIC AGENTS	Estrogens

ESTRADIOL VALERATE 20 MG/ML INTRAMUSCULAR OIL	ESTROGENIC AGENTS	Estrogens
ESTRADIOL VALERATE 40 MG/ML INTRAMUSCULAR OIL	ESTROGENIC AGENTS	Estrogens
ESTRADIOL VALERATE IM	ESTROGENIC AGENTS	Estrogens
ESTRADIOL-LEVONORGESTREL TD	ESTROGENIC AGENTS	Estrogen-Progestin
ESTRADIOL-NORETHINDRONE ACET 0.5 MG-0.1 MG TABLET	ESTROGENIC AGENTS	Estrogen-Progestin
ESTRADIOL-NORETHINDRONE ACET 1 MG-0.5 MG TABLET	ESTROGENIC AGENTS	Estrogen-Progestin
ESTRADIOL-NORETHINDRONE ACET ORAL	ESTROGENIC AGENTS	Estrogen-Progestin
ESTRADIOL-NORETHINDRONE ACET TD	ESTROGENIC AGENTS	Estrogen-Progestin
ESTRADIOL-NORGESTIMATE 1 MG (15)/1 MG-0.09 MG (15) TABLET	ESTROGENIC AGENTS	Estrogen-Progestin
ESTRADIOL-NORGESTIMATE ORAL	ESTROGENIC AGENTS	Estrogen-Progestin
ESTRASORB 2.5 MG/GRAM (1.74 GRAM) TRANSDERMAL EMULSION PACKET	ESTROGENIC AGENTS	Estrogens
ESTRASORB TD	ESTROGENIC AGENTS	Estrogens
ESTROGEL 1.25 GRAM/ACTUATION (0.06%) TRANSDERMAL GEL PUMP	ESTROGENIC AGENTS	Estrogens
ESTROGEL TD	ESTROGENIC AGENTS	Estrogens
ESTROGENS CONJ-MEPROBAMATE 0.45 MG-200 MG TABLET	ESTROGENIC AGENTS	Estrogen-Antianxiety Agent
ESTROGENS CONJ-MEPROBAMATE ORAL	ESTROGENIC AGENTS	Estrogen-Antianxiety Agent
ESTROGENS, CONJUGATED, SYNTHETIC A 0.3 MG TABLET	ESTROGENIC AGENTS	Estrogens
ESTROGENS, CONJUGATED, SYNTHETIC A 0.45 MG TABLET	ESTROGENIC AGENTS	Estrogens
ESTROGENS, CONJUGATED, SYNTHETIC A 0.625 MG TABLET	ESTROGENIC AGENTS	Estrogens
ESTROGENS, CONJUGATED, SYNTHETIC A 0.9 MG TABLET	ESTROGENIC AGENTS	Estrogens
ESTROGENS, CONJUGATED, SYNTHETIC A 1.25 MG TABLET	ESTROGENIC AGENTS	Estrogens
ESTROGENS, CONJUGATED, SYNTHETIC B 0.3 MG TABLET	ESTROGENIC AGENTS	Estrogens
ESTROGENS, CONJUGATED, SYNTHETIC B 0.45 MG TABLET	ESTROGENIC AGENTS	Estrogens
ESTROGENS, CONJUGATED, SYNTHETIC B 0.625 MG TABLET	ESTROGENIC AGENTS	Estrogens
ESTROGENS, CONJUGATED, SYNTHETIC B 0.9 MG TABLET	ESTROGENIC AGENTS	Estrogens
ESTROGENS, CONJUGATED, SYNTHETIC B 1.25 MG TABLET	ESTROGENIC AGENTS	Estrogens
ESTROPIPATE 0.75 MG TABLET	ESTROGENIC AGENTS	Estrogens
ESTROPIPATE 1.5 MG TABLET	ESTROGENIC AGENTS	Estrogens
ESTROPIPATE 3 MG TABLET	ESTROGENIC AGENTS	Estrogens
ESTROPIPATE ORAL	ESTROGENIC AGENTS	Estrogens
ETHINYL ESTRADIOL 0.05 MG TABLET	ESTROGENIC AGENTS	Estrogens
ETHINYL ESTRADIOL 0.5 MG TABLET	ESTROGENIC AGENTS	Estrogens
ETHINYL ESTRADIOL ORAL	ESTROGENIC AGENTS	Estrogens
EVAMIST 1.53 MG/SPRAY (1.7 %) TRANSDERMAL SPRAY	ESTROGENIC AGENTS	Estrogens
EVAMIST TD	ESTROGENIC AGENTS	Estrogens
FEMHRT 1/5 1 MG-5 MCG TABLET	ESTROGENIC AGENTS	Estrogen-Progestin
FEMHRT 1/5 ORAL	ESTROGENIC AGENTS	Estrogen-Progestin
FEMHRT LOW DOSE 0.5 MG-2.5 MCG TABLET	ESTROGENIC AGENTS	Estrogen-Progestin
FEMHRT LOW DOSE ORAL	ESTROGENIC AGENTS	Estrogen-Progestin
FYAVOLV 0.5 MG-2.5 MCG TABLET	ESTROGENIC AGENTS	Estrogen-Progestin
FYAVOLV 1 MG-5 MCG TABLET	ESTROGENIC AGENTS	Estrogen-Progestin
FYAVOLV ORAL	ESTROGENIC AGENTS	Estrogen-Progestin
JEVANTIQUE LO 0.5 MG-2.5 MCG TABLET	ESTROGENIC AGENTS	Estrogen-Progestin
JINTELI 1 MG-5 MCG TABLET	ESTROGENIC AGENTS	Estrogen-Progestin
JINTELI ORAL	ESTROGENIC AGENTS	Estrogen-Progestin
LOPREEZA 0.5 MG-0.1 MG TABLET	ESTROGENIC AGENTS	Estrogen-Progestin
LOPREEZA 1 MG-0.5 MG TABLET	ESTROGENIC AGENTS	Estrogen-Progestin
LOPREEZA ORAL	ESTROGENIC AGENTS	Estrogen-Progestin
LYLLANA 0.025 MG/24 HR TRANSDERMAL PATCH	ESTROGENIC AGENTS	Estrogens
LYLLANA 0.05 MG/24 HR TRANSDERMAL PATCH	ESTROGENIC AGENTS	Estrogens
LYLLANA 0.075 MG/24 HR TRANSDERMAL PATCH	ESTROGENIC AGENTS	Estrogens

LYLLANA 0.1 MG/24 HR TRANSDERMAL PATCH	ESTROGENIC AGENTS	Estrogens
MENEST 0.3 MG TABLET	ESTROGENIC AGENTS	Estrogens
MENEST 0.625 MG TABLET	ESTROGENIC AGENTS	Estrogens
MENEST 1.25 MG TABLET	ESTROGENIC AGENTS	Estrogens
MENEST ORAL	ESTROGENIC AGENTS	Estrogens
MENOSTAR 14 MCG/24 HR TRANSDERMAL PATCH	ESTROGENIC AGENTS	Estrogens
MENOSTAR TD	ESTROGENIC AGENTS	Estrogens
MIMVEY 1 MG-0.5 MG TABLET	ESTROGENIC AGENTS	Estrogen-Progestin
MIMVEY LO 0.5 MG-0.1 MG TABLET	ESTROGENIC AGENTS	Estrogen-Progestin
MIMVEY LO ORAL	ESTROGENIC AGENTS	Estrogen-Progestin
MIMVEY ORAL	ESTROGENIC AGENTS	Estrogen-Progestin
MINIVELLE 0.025 MG/24 HR TRANSDERMAL PATCH	ESTROGENIC AGENTS	Estrogens
MINIVELLE 0.0375 MG/24 HR TRANSDERMAL PATCH	ESTROGENIC AGENTS	Estrogens
MINIVELLE 0.05 MG/24 HR TRANSDERMAL PATCH	ESTROGENIC AGENTS	Estrogens
MINIVELLE 0.075 MG/24 HR TRANSDERMAL PATCH	ESTROGENIC AGENTS	Estrogens
MINIVELLE 0.1 MG/24 HR TRANSDERMAL PATCH	ESTROGENIC AGENTS	Estrogens
MINIVELLE TD	ESTROGENIC AGENTS	Estrogens
NORETHINDRONE AC-ETH ESTRADIOL ORAL	ESTROGENIC AGENTS	Estrogen-Progestin
NORETHINDRONE ACETATE 0.5 MG-ETHINYL ESTRADIOL 2.5 MCG TABLET	ESTROGENIC AGENTS	Estrogen-Progestin
NORETHINDRONE ACETATE 1 MG-ETHINYL ESTRADIOL 5 MCG TABLET	ESTROGENIC AGENTS	Estrogen-Progestin
OGEN 0.625 ORAL	ESTROGENIC AGENTS	Estrogens
OGEN 1.25 ORAL	ESTROGENIC AGENTS	Estrogens
ORTHO-PREFEST ORAL	ESTROGENIC AGENTS	Estrogen-Progestin
PREFEST 1 MG (15)/1 MG-0.09 MG (15) TABLET	ESTROGENIC AGENTS	Estrogen-Progestin
PREFEST ORAL	ESTROGENIC AGENTS	Estrogen-Progestin
PREMARIN 0.3 MG TABLET	ESTROGENIC AGENTS	Estrogens
PREMARIN 0.45 MG TABLET	ESTROGENIC AGENTS	Estrogens
PREMARIN 0.625 MG TABLET	ESTROGENIC AGENTS	Estrogens
PREMARIN 0.9 MG TABLET	ESTROGENIC AGENTS	Estrogens
PREMARIN 1.25 MG TABLET	ESTROGENIC AGENTS	Estrogens
PREMARIN ORAL	ESTROGENIC AGENTS	Estrogens
PREMPHASE 0.625 MG(14)/0.625 MG-5MG(14) TABLET	ESTROGENIC AGENTS	Estrogen-Progestin
PREMPHASE ORAL	ESTROGENIC AGENTS	Estrogen-Progestin
PREMPRO 0.3 MG-1.5 MG TABLET	ESTROGENIC AGENTS	Estrogen-Progestin
PREMPRO 0.45 MG-1.5 MG TABLET	ESTROGENIC AGENTS	Estrogen-Progestin
PREMPRO 0.625 MG-2.5 MG TABLET	ESTROGENIC AGENTS	Estrogen-Progestin
PREMPRO 0.625 MG-5 MG TABLET	ESTROGENIC AGENTS	Estrogen-Progestin
PREMPRO ORAL	ESTROGENIC AGENTS	Estrogen-Progestin
SYNTHETIC CONJ ESTROGENS A ORAL	ESTROGENIC AGENTS	Estrogens
SYNTHETIC CONJ ESTROGENS B ORAL	ESTROGENIC AGENTS	Estrogens
VIVELLE 0.075 MG/24 HR TRANSDERMAL PATCH	ESTROGENIC AGENTS	Estrogens
VIVELLE 0.1 MG/24 HR TRANSDERMAL PATCH	ESTROGENIC AGENTS	Estrogens
VIVELLE TD	ESTROGENIC AGENTS	Estrogens
VIVELLE-DOT 0.025 MG/24 HR TRANSDERMAL PATCH	ESTROGENIC AGENTS	Estrogens
VIVELLE-DOT 0.0375 MG/24 HR TRANSDERMAL PATCH	ESTROGENIC AGENTS	Estrogens
VIVELLE-DOT 0.05 MG/24 HR TRANSDERMAL PATCH	ESTROGENIC AGENTS	Estrogens
VIVELLE-DOT 0.075 MG/24 HR TRANSDERMAL PATCH	ESTROGENIC AGENTS	Estrogens
VIVELLE-DOT 0.1 MG/24 HR TRANSDERMAL PATCH	ESTROGENIC AGENTS	Estrogens
VIVELLE-DOT TD	ESTROGENIC AGENTS	Estrogens
AYGESTIN 5 MG TABLET	PROGESTATIONAL AGENTS	Progestins
AYGESTIN ORAL	PROGESTATIONAL AGENTS	Progestins
CRINONE 4 % VAGINAL GEL	PROGESTATIONAL AGENTS	Vaginal Progestins
CRINONE VAGINAL	PROGESTATIONAL AGENTS	Vaginal Progestins
DEPO-PROVERA IM	PROGESTATIONAL AGENTS	Antineoplastic - Progestins
HYDROXYPROGESTERONE CAPROATE 250 MG/ML INTRAMUSCULAR OIL	PROGESTATIONAL AGENTS	Progestins

HYDROXYPROGESTERONE CAPROATE 250 MG/ML INTRAMUSCULAR SYRINGE	PROGESTATIONAL AGENTS	Progestins
HYDROXYPROGESTERONE CAPROATE IM	PROGESTATIONAL AGENTS	Progestins
MEDROXYPROGESTERONE 10 MG TABLET	PROGESTATIONAL AGENTS	Progestins
MEDROXYPROGESTERONE 2.5 MG TABLET	PROGESTATIONAL AGENTS	Progestins
MEDROXYPROGESTERONE 400 MG/ML INTRAMUSCULAR SUSPENSION	PROGESTATIONAL AGENTS	Antineoplastic - Progestins
MEDROXYPROGESTERONE 5 MG TABLET	PROGESTATIONAL AGENTS	Progestins
MEDROXYPROGESTERONE IM	PROGESTATIONAL AGENTS	Antineoplastic - Progestins
MEDROXYPROGESTERONE ORAL	PROGESTATIONAL AGENTS	Progestins
NORETHINDRONE ACETATE 5 MG TABLET	PROGESTATIONAL AGENTS	Progestins
NORETHINDRONE ACETATE ORAL	PROGESTATIONAL AGENTS	Progestins
PROCHIEVE VAGINAL	PROGESTATIONAL AGENTS	Vaginal Progestins
PROGESTERONE	PROGESTATIONAL AGENTS	Progestins
PROGESTERONE 50 MG/ML INTRAMUSCULAR OIL	PROGESTATIONAL AGENTS	Progestins
PROGESTERONE ACETATE (1:1) 50 MG/ML (SKIN TEST)	PROGESTATIONAL AGENTS	Progestins
PROGESTERONE IM	PROGESTATIONAL AGENTS	Progestins
PROGESTERONE IN OIL 50 MG/ML INTRAMUSCULAR	PROGESTATIONAL AGENTS	Progestins
PROGESTERONE IN OIL IM	PROGESTATIONAL AGENTS	Progestins
PROGESTERONE MICRONIZED 10 % TRANSDERMAL CREAM	PROGESTATIONAL AGENTS	Progestins
PROGESTERONE MICRONIZED 100 MG CAPSULE	PROGESTATIONAL AGENTS	Progestins
PROGESTERONE MICRONIZED 200 MG CAPSULE	PROGESTATIONAL AGENTS	Progestins
PROGESTERONE MICRONIZED 4 % VAGINAL GEL	PROGESTATIONAL AGENTS	Vaginal Progestins
PROGESTERONE MICRONIZED ORAL	PROGESTATIONAL AGENTS	Progestins
PROGESTERONE MICRONIZED TD	PROGESTATIONAL AGENTS	Progestins
PROGESTERONE MICRONIZED VAGINAL	PROGESTATIONAL AGENTS	Vaginal Progestins
PROMETRIUM 100 MG CAPSULE	PROGESTATIONAL AGENTS	Progestins
PROMETRIUM 200 MG CAPSULE	PROGESTATIONAL AGENTS	Progestins
PROMETRIUM ORAL	PROGESTATIONAL AGENTS	Progestins
PROVERA 10 MG TABLET	PROGESTATIONAL AGENTS	Progestins
PROVERA 2.5 MG TABLET	PROGESTATIONAL AGENTS	Progestins
PROVERA 5 MG TABLET	PROGESTATIONAL AGENTS	Progestins
PROVERA ORAL	PROGESTATIONAL AGENTS	Progestins

Supplementary Table 2: Contraceptive class mapping from medication name
Medication names mapped to one of six modalities considered – Oral, Implant, Intrauterine device (IUD), Injection (intramuscular or subcutaneous), Transdermal, and Intravaginal.

Search string / Medication name	Pharmaceutical Subclass	Mapped value
etonogestreL 68 mg subdermal implant	Contraceptive Implant - Progestin	Implant
levonorgestreL 21 mcg/24 hours (8 yrs) 52 mg intrauterine device	Contraceptive Intrauterine - Progesterone IUD	Intrauterine
drospirenone 3 mg-ethinyl estradioL 0.03 mg tablet	Contraceptive Oral - Monophasic	Oral
levonorgestreL-ethinyl estradioL 0.1 mg-20 mcg tablet	Contraceptive Oral - Monophasic	Oral
norethindrone 1 mg-ethinyl estradioL 20 mcg (21)-iron 75 mg (7) tablet	Contraceptive Oral - Monophasic	Oral
copper 380 square mm intrauterine device	Contraceptive Intrauterine - Copper IUD	Intrauterine
levonorgestreL 0.15 mg-ethinyl estradiol 30 mcg tablets,3 mos pack(91)	Contraceptive Oral - Monophasic	Oral
etonogestreL 0.12 mg-ethinyl estradioL 0.015 mg/24 hr vaginal ring	Contraceptives - Intravaginal, Systemic - Estrogen and Progestin Comb.	Intravaginal
levonorgestreL 0.15 mg-ethinyl estradiol 0.03 mg tablet	Contraceptive Oral - Monophasic	Oral
norethindrone (contraceptive) 0.35 mg tablet	Contraceptive Oral - Progestin	Oral
drospirenone 3 mg-ethinyl estradioL 0.02 mg tablet	Contraceptive Oral - Monophasic	Oral
drospirenone-ethinyl estradiol oral	Contraceptive Oral - Monophasic	Oral
norelgestromin 150 mcg-e.estradioL 35 mcg/24 hr weekly transderm patch	Contraceptive Transdermal Combinations - Estrogen and Progestin Comb.	Transdermal
norethindrone 1.5 mg-ethinyl estradioL 30 mcg(21)/iron 75 mg(7) tablet	Contraceptive Oral - Monophasic	Oral
norgestimate-ethinyl estradioL 0.18 mg/0.215mg/0.25mg-35 mcg(28)tablet	Contraceptive Oral - Triphasic	Oral
levonorgestreL 14 mcg/24 hrs (3 yrs) 13.5 mg intrauterine device	Contraceptive Intrauterine - Progesterone IUD	Intrauterine
segesterone acet 0.15 mg-ethinyl estradioL 0.013 mg/24 hr vaginal ring	Contraceptives - Intravaginal, Systemic - Estrogen and Progestin Comb.	Intravaginal
norethindrone acetate 1 mg-ethinyl estradioL 20 mcg tablet	Contraceptive Oral - Monophasic	Oral
levonorgestreL 17.5 mcg/24 hrs (5yrs) 19.5mg intrauterine device	Contraceptive Intrauterine - Progesterone IUD	Intrauterine
norgestrel 0.3 mg-ethinyl estradioL 30 mcg tablet	Contraceptive Oral - Monophasic	Oral
norethindrone 1 mg-ethinyl estradioL 10 mcg (24)-iron 10 mcg(2) tablet	Contraceptive Oral - Biphasic	Oral
desogestreL 0.15 mg-ethinyl estradioL 0.03 mg tablet	Contraceptive Oral - Monophasic	Oral
norethindrone acetate 1.5 mg-ethinyl estradioL 30 mcg tablet	Contraceptive Oral - Monophasic	Oral
norethindrone 0.5 mg-ethinyl estradioL 35 mcg tablet	Contraceptive Oral - Monophasic	Oral
norgestimate 0.25 mg-ethinyl estradioL 35 mcg tablet	Contraceptive Oral - Monophasic	Oral
levonorgestrel-ethinyl estradiol transdermal	Contraceptive Transdermal Combinations - Estrogen and Progestin Comb.	Transdermal
intrauterine device (IUD)	Medical Supplies and DME - IUDs	Intrauterine
norgestimate 0.18 mg/0.215 mg/0.25 mg-ethinyl estradioL 25 mcg tablet	Contraceptive Oral - Triphasic	Oral
L norgest/E estradiol-E estrad 0.15 mg-30 mcg (84)/10 mcg(7) tabs,3mos	Contraceptive Oral - Biphasic	Oral
levonorgestreL 20.4 mcg/24 hrs (8 yrs) 52 mg intrauterine device	Contraceptive Intrauterine - Progesterone IUD	Intrauterine
medroxyPROGESTERone 150 mg/mL intramuscular syringe	Contraceptive Injectable - Progestin	Injectable
medroxyPROGESTERone 150 mg/mL intramuscular suspension	Contraceptive Injectable - Progestin	Injectable
norethindrone 1 mg-e. estradioL 20 mcg (24)-iron 75 mg (4) chew tablet	Contraceptive Oral - Monophasic	Oral
levonorgestreL-ethinyl estradioL 90 mcg-20 mcg (28) tablet	Contraceptive Oral - Monophasic	Oral
norethindrone 1 mg-ethinyl estradioL 20 mcg (24)-iron 75 mg (4) tablet	Contraceptive Oral - Monophasic	Oral
estradiol-dienogest 3 mg/2 mg-2 mg/2 mg-3 mg/1 mg tablet	Contraceptive Oral - Quadraphasic	Oral
norethindrone 1 mg-ethinyl estradioL 35 mcg tablet	Contraceptive Oral - Monophasic	Oral
drospirenone (contraceptive) 4 mg (28) tablet	Contraceptive Oral - Progestin	Oral
etonogestrel subdermal	Contraceptive Implant - Progestin	Implant
levonorgestrel intrauterine	Contraceptive Intrauterine - Progesterone IUD	Intrauterine
norethindrone-eth. estradiol-iron 1-20 (5)/1-30(7)/1mg-35mcg(9) tablet	Contraceptive Oral - Triphasic	Oral
L norgest/E estradiol-E estrad 0.1 mg-20 mcg (84)/10 mcg (7) tabs,3mos	Contraceptive Oral - Biphasic	Oral
norethindrone-e.estradiol triphasic 0.5 mg/0.75 mg/1 mg-35 mcg tablet	Contraceptive Oral - Triphasic	Oral
norethindrone 0.4 mg-ethinyl estradioL 35 mcg tablet	Contraceptive Oral - Monophasic	Oral
desogestreL-e.estradioL 0.15 mg-0.02 mg(21)/e.estrad 0.01 mg(5) tablet	Contraceptive Oral - Biphasic	Oral
levonorgestrel/ethinyl estradiol and ethinyl estradiol oral	Contraceptive Oral - Biphasic	Oral
desogestrel-ethinyl estradiol oral	Contraceptive Oral - Monophasic	Oral
norgestimate-ethinyl estradiol oral	Contraceptive Oral - Monophasic	Oral

desogestreL-ethinyl estradioL 0.1 mg/0.125 mg/0.15 mg-25 mcg tablet	Contraceptive Oral - Triphasic	Oral
ethynodiol diacetate-ethinyl estradioL 1 mg-35 mcg tablet	Contraceptive Oral - Monophasic	Oral
norethin-ethinyl estradioL-iron 0.4 mg-35 mcg(21)/75 mg(7) chew tablet	Contraceptive Oral - Monophasic	Oral
norgestrel 0.5 mg-ethinyl estradioL 50 mcg tablet	Contraceptive Oral - Monophasic	Oral
norethindrone 1 mg-ethinyl estradioL 35 mcg (21) tablet	Contraceptive Oral - Monophasic	Oral
levonorgestreL 120 mcg-e.estradiol 30 mcg/24 hr weekly transderm patch	Contraceptive Transdermal Combinations - Estrogen and Progestin Comb.	Transdermal
drospiren-e.estrad-l.mefoL 3 mg-0.02 mg-0.451 mg(24)/0.451 mg(4)tablet	Contraceptive Oral - Monophasic	Oral
ethynodiol diacetate-ethinyl estradioL 1 mg-50 mcg tablet	Contraceptive Oral - Monophasic	Oral
l.norgest-eth.estradiol triphasic 50-30 (6)/75-40(5)/125-30(10) tablet	Contraceptive Oral - Triphasic	Oral
norethindrone acetate-ethinyl estradiol-iron oral	Contraceptive Oral - Biphasic	Oral
levonorgestreL 0.1 mg-eth.estradiol 0.02 mg(21)/iron 36.5 mg(7) tablet	Contraceptive Oral - Monophasic	Oral
norethin-ethinyl estradioL-iron 0.8 mg-25 mcg(24)/75 mg(4) chew tablet	Contraceptive Oral - Monophasic	Oral
medroxyPROGESTERone 104 mg/0.65 mL subcutaneous syringe	Contraceptive Injectable - Progestin	Injectable
copper intrauterine	Contraceptive Intrauterine - Copper IUD	Intrauterine
levonorgestrel-ethinyl estradiol oral	Contraceptive Oral - Monophasic	Oral
norethindrone (contraceptive) oral	Contraceptive Oral - Progestin	Oral
norethindrone-ethinyl estradiol triphasic oral	Contraceptive Oral - Triphasic	Oral
drospirenone (contraceptive) oral	Contraceptive Oral - Progestin	Oral
etonogestrel-ethinyl estradiol vaginal	Contraceptives - Intravaginal, Systemic - Estrogen and Progestin Comb.	Intravaginal
norethindrone 1 mg-ethin. estradioL 20 mcg (24)-iron 75 mg (4) capsule	Contraceptive Oral - Monophasic	Oral
norethindrone acetate-ethinyl estradiol oral	Contraceptive Oral - Monophasic	Oral
L norgest/EE 0.15-0.02mg/0.15-0.025mg/0.15-0.03mg/EE 0.01 mg tabs,3mo	Contraceptive Oral - Quadraphasic	Oral
levonorgestrel implant	Contraceptive Implant - Progestin	Implant
desogestrel-ethinyl estradiol/ethiny estradiol oral	Contraceptive Oral - Biphasic	Oral
norethindrone-ethinyl estradiol-iron oral	Contraceptive Oral - Monophasic	Oral
drospiren-e.estrad-l.mefoL 3 mg-0.03 mg-0.451 mg(21)/0.451 mg(7)tablet	Contraceptive Oral - Monophasic	Oral
norgestrel-ethinyl estradiol oral	Contraceptive Oral - Monophasic	Oral
norethindrone-ethinyl estradioL triphasic 0.5/1/0.5 mg-35 mcg tablet	Contraceptive Oral - Triphasic	Oral
drospirenone 3 mg-estetrol 14.2 mg (28) tablet	Contraceptive Oral - Monophasic	Oral
segesterone acetate-ethinyl estradiol vaginal	Contraceptives - Intravaginal, Systemic - Estrogen and Progestin Comb.	Intravaginal
ethynodiol diacetate-ethinyl estradiol oral	Contraceptive Oral - Monophasic	Oral
levonorgestreL 0.1 mg-ethinyl estradiol 20 mcg chewable tablet	Contraceptive Oral - Monophasic	Oral
l.norgest-eth.estradiol triphasic oral	Contraceptive Oral - Triphasic	Oral
norethindrone-ethinyl estradiol oral	Contraceptive Oral - Monophasic	Oral
estradiol-dienogest oral	Contraceptive Oral - Quadraphasic	Oral
norelgestromin-ethinyl estradiol transdermal	Contraceptive Transdermal Combinations - Estrogen and Progestin Comb.	Transdermal
norethindrone-mestranol oral	Contraceptive Oral - Monophasic	Oral
drospirenone-estetrol oral	Contraceptive Oral - Monophasic	Oral
norgestrel oral	Contraceptive Oral - Progestin	Oral
levonorgestrel-ethinyl estradiol-iron oral	Contraceptive Oral - Monophasic	Oral
drospirenone-ethinyl estradiol-levomefolate oral	Contraceptive Oral - Monophasic	Oral
norethindrone-ethinyl estradiol biphasic oral	Contraceptive Oral - Biphasic	Oral
medroxyprogesterone subcutaneous	Contraceptive Injectable - Progestin	Injectable
norethindrone 1 mg-mestranoL 50 mcg tablet	Contraceptive Oral - Monophasic	Oral
norethin-eth estrad biphasic 0.5 mg-35 mcg(10)/1 mg-35 mcg(11) tablet	Contraceptive Oral - Biphasic	Oral
norethindrone-ethinyl estradioL 1 mg-50 mcg tablet	Contraceptive Oral - Monophasic	Oral
norethindrone 1 mg-e.estradioL 10 mcg(24)-iron 10 mg(2)chew tab,tablet	Contraceptive Oral - Monophasic	Oral
progesterone intrauterine	Contraceptive Intrauterine - Progesterone IUD	Intrauterine
norgestimate-ethinyl estradioL 0.18 mg/0.215mg/0.25mg-35 mcg(21)tablet	Contraceptive Oral - Triphasic	Oral
norethindrone-ethinyl estradioL biphasic 0.5-35/1-35 mg-mcg (7) tablet	Contraceptive Oral - Biphasic	Oral
norgestrel 0.075 mg tablet	Contraceptive Oral - Progestin	Oral
ETONOGESTREL 68 MG SUBDERMAL IMPLANT	Contraceptive Implant - Progestin	Implant
LEVONORGESTREL 21 MCG/24 HOURS (8 YRS) 52 MG INTRAUTERINE DEVICE	Contraceptive Intrauterine - Progesterone IUD	Intrauterine
DROSPIRENONE 3 MG-ETHINYL ESTRADIOL 0.03 MG TABLET	Contraceptive Oral - Monophasic	Oral

VIENVA 0.1 MG-20 MCG TABLET	Contraceptive Oral - Monophasic	Oral
BLISOVI FE 1/20 (28) 1 MG-20 MCG (21)/75 MG (7) TABLET	Contraceptive Oral - Monophasic	Oral
HAILEY FE 1/20 (28) 1 MG-20 MCG (21)/75 MG (7) TABLET	Contraceptive Oral - Monophasic	Oral
PARAGARD T 380A 380 SQUARE MM INTRAUTERINE DEVICE	Contraceptive Intrauterine - Copper IUD	Intrauterine
LEVONORGESTREL 0.15 MG-ETHINYL ESTRADIOL 30 MCG TABLETS,3 MOS PACK(91)	Contraceptive Oral - Monophasic	Oral
ETONOGESTREL 0.12 MG-ETHINYL ESTRADIOL 0.015 MG/24 HR VAGINAL RING	Contraceptives - Intravaginal, Systemic - Estrogen and Progestin Comb.	Intravaginal
LEVONORGESTREL 0.15 MG-ETHINYL ESTRADIOL 0.03 MG TABLET	Contraceptive Oral - Monophasic	Oral
NORETHINDRONE (CONTRACEPTIVE) 0.35 MG TABLET	Contraceptive Oral - Progestin	Oral
DROSPIRENONE 3 MG-ETHINYL ESTRADIOL 0.02 MG TABLET	Contraceptive Oral - Monophasic	Oral
YAZ (28) ORAL	Contraceptive Oral - Monophasic	Oral
NORELGESTROMIN 150 MCG-E.ESTRADIOL 35 MCG/24 HR WEEKLY TRANSDERM PATCH	Contraceptive Transdermal Combinations - Estrogen and Progestin Comb.	Transdermal
COPPER 380 SQUARE MM INTRAUTERINE DEVICE	Contraceptive Intrauterine - Copper IUD	Intrauterine
LEVONORGESTREL-ETHINYL ESTRADIOL 0.1 MG-20 MCG TABLET	Contraceptive Oral - Monophasic	Oral
NORETHINDRONE 1.5 MG-ETHINYL ESTRADIOL 30 MCG(21)/IRON 75 MG(7) TABLET	Contraceptive Oral - Monophasic	Oral
NORGESTIMATE-ETHINYL ESTRADIOL 0.18 MG/0.215MG/0.25MG-35 MCG(28)TABLET	Contraceptive Oral - Triphasic	Oral
SKYLA 14 MCG/24 HRS (3 YRS) 13.5 MG INTRAUTERINE DEVICE	Contraceptive Intrauterine - Progesterone IUD	Intrauterine
ANNOVERA 0.15 MG-0.013 MG/24 HR VAGINAL RING	Contraceptives - Intravaginal, Systemic - Estrogen and Progestin Comb.	Intravaginal
NORETHINDRONE ACETATE 1 MG-ETHINYL ESTRADIOL 20 MCG TABLET	Contraceptive Oral - Monophasic	Oral
MIRENA 21 MCG/24 HOURS (8 YRS) 52 MG INTRAUTERINE DEVICE	Contraceptive Intrauterine - Progesterone IUD	Intrauterine
KYLEENA 17.5 MCG/24 HRS (5YRS) 19.5MG INTRAUTERINE DEVICE	Contraceptive Intrauterine - Progesterone IUD	Intrauterine
NORGESTREL 0.3 MG-ETHINYL ESTRADIOL 30 MCG TABLET	Contraceptive Oral - Monophasic	Oral
LO LOESTRIN FE 1 MG-10 MCG (24)/10 MCG (2) TABLET	Contraceptive Oral - Biphasic	Oral
ENSKYCE 0.15 MG-0.03 MG TABLET	Contraceptive Oral - Monophasic	Oral
YASMIN (28) 3 MG-0.03 MG TABLET	Contraceptive Oral - Monophasic	Oral
TRI-LINYAH (28) 0.18 MG(7)/0.215 MG(7)/0.25 MG(7)-35 MCG TABLET	Contraceptive Oral - Triphasic	Oral
NORETHINDRONE 1 MG-ETHINYL ESTRADIOL 20 MCG (21)-IRON 75 MG (7) TABLET	Contraceptive Oral - Monophasic	Oral
JUNEL 1.5/30 (21) 1.5 MG-30 MCG TABLET	Contraceptive Oral - Monophasic	Oral
ISIBLOOM 0.15 MG-0.03 MG TABLET	Contraceptive Oral - Monophasic	Oral
NORTREL 0.5/35 (28) 0.5 MG-35 MCG TABLET	Contraceptive Oral - Monophasic	Oral
NORGESTIMATE 0.25 MG-ETHINYL ESTRADIOL 35 MCG TABLET	Contraceptive Oral - Monophasic	Oral
TWIRLA TD	Contraceptive Transdermal Combinations - Estrogen and Progestin Comb.	Transdermal
INTRAUTERINE DEVICE (IUD) INTRAUTERINE	Medical Supplies and DME - IUDs	Intrauterine
NORGESTIMATE 0.18 MG/0.215 MG/0.25 MG-ETHINYL ESTRADIOL 25 MCG TABLET	Contraceptive Oral - Triphasic	Oral
L NORGEST/E ESTRADIOL-E ESTRAD 0.15 MG-30 MCG (84)/10 MCG(7) TABS,3MOS	Contraceptive Oral - Biphasic	Oral
LEVONORGESTREL 20.4 MCG/24 HRS (8 YRS) 52 MG INTRAUTERINE DEVICE	Contraceptive Intrauterine - Progesterone IUD	Intrauterine
MEDROXYPROGESTERONE 150 MG/ML INTRAMUSCULAR SYRINGE	Contraceptive Injectable - Progestin	Injectable
LEVONORGESTREL 17.5 MCG/24 HRS (5YRS) 19.5MG INTRAUTERINE DEVICE	Contraceptive Intrauterine - Progesterone IUD	Intrauterine
MEDROXYPROGESTERONE 150 MG/ML INTRAMUSCULAR SUSPENSION	Contraceptive Injectable - Progestin	Injectable
NUVARING 0.12 MG-0.015 MG/24 HR VAGINAL	Contraceptives - Intravaginal, Systemic - Estrogen and Progestin Comb.	Intravaginal
ZAFEMY 150 MCG-35 MCG/24 HR TRANSDERMAL PATCH	Contraceptive Transdermal Combinations - Estrogen and Progestin Comb.	Transdermal
LUTERA (28) 0.1 MG-20 MCG TABLET	Contraceptive Oral - Monophasic	Oral
NORETHINDRONE 1 MG-E. ESTRADIOL 20 MCG (24)-IRON 75 MG (4) CHEW TABLET	Contraceptive Oral - Monophasic	Oral
LEVONORGESTREL-ETHINYL ESTRADIOL 90 MCG-20 MCG (28) TABLET	Contraceptive Oral - Monophasic	Oral
ELINEST 0.3 MG-30 MCG TABLET	Contraceptive Oral - Monophasic	Oral
JUNEL FE 1/20 (28) 1 MG-20 MCG (21)/75 MG (7) TABLET	Contraceptive Oral - Monophasic	Oral
MONO-LINYAH 0.25 MG-35 MCG TABLET	Contraceptive Oral - Monophasic	Oral
LORYNA (28) 3 MG-0.02 MG TABLET	Contraceptive Oral - Monophasic	Oral
JUNEL FE 1.5/30 (28) 1.5 MG-30 MCG (21)/75 MG (7) TABLET	Contraceptive Oral - Monophasic	Oral
MARLISSA (28) 0.15 MG-0.03 MG TABLET	Contraceptive Oral - Monophasic	Oral
LARIN 24 FE 1 MG-20 MCG (24)/75 MG (4) TABLET	Contraceptive Oral - Monophasic	Oral
NATAZIA 3 MG/2 MG-2 MG/2 MG-3 MG/1 MG TABLET	Contraceptive Oral - Quadraphasic	Oral
DAYSEE 0.15 MG-30 MCG (84)/10 MCG(7) TABLETS,3 MONTH DOSE PACK	Contraceptive Oral - Biphasic	Oral

NIKKI (28) 3 MG-0.02 MG TABLET	Contraceptive Oral - Monophasic	Oral
NORTREL 1/35 (28) 1 MG-35 MCG TABLET	Contraceptive Oral - Monophasic	Oral
TRI-LO-ESTARYLLA 0.18 MG/0.215 MG/0.25 MG-25 MCG TABLET	Contraceptive Oral - Triphasic	Oral
HEATHER 0.35 MG TABLET	Contraceptive Oral - Progestin	Oral
NORA-BE 0.35 MG TABLET	Contraceptive Oral - Progestin	Oral
CRYSELLE (28) 0.3 MG-30 MCG TABLET	Contraceptive Oral - Monophasic	Oral
JENCYCLA 0.35 MG TABLET	Contraceptive Oral - Progestin	Oral
SLYND 4 MG (28) TABLET	Contraceptive Oral - Progestin	Oral
NEXPLANON SUBDERMAL	Contraceptive Implant - Progestin	Implant
NEXPLANON 68 MG SUBDERMAL IMPLANT	Contraceptive Implant - Progestin	Implant
KYLEENA INTRAUTERINE	Contraceptive Intrauterine - Progesterone IUD	Intrauterine
LILETTA 20.4 MCG/24 HRS (8 YRS) 52 MG INTRAUTERINE DEVICE	Contraceptive Intrauterine - Progesterone IUD	Intrauterine
NORETHINDRONE-ETH. ESTRADIOL-IRON 1-20 (5)/1-30(7)/1MG-35MCG(9) TABLET	Contraceptive Oral - Triphasic	Oral
L NORGEST/E ESTRADIOL-E ESTRAD 0.1 MG-20 MCG (84)/10 MCG (7) TABS,3MOS	Contraceptive Oral - Biphasic	Oral
DESOGESTREL 0.15 MG-ETHINYL ESTRADIOL 0.03 MG TABLET	Contraceptive Oral - Monophasic	Oral
NORETHINDRONE 0.5 MG-ETHINYL ESTRADIOL 35 MCG TABLET	Contraceptive Oral - Monophasic	Oral
NORETHINDRONE-E.ESTRADIOL TRIPHASIC 0.5 MG/0.75 MG/1 MG-35 MCG TABLET	Contraceptive Oral - Triphasic	Oral
NORETHINDRONE 0.4 MG-ETHINYL ESTRADIOL 35 MCG TABLET	Contraceptive Oral - Monophasic	Oral
DESOGESTREL-E.ESTRADIOL 0.15 MG-0.02 MG(21)/E.ESTRAD 0.01 MG(5) TABLET	Contraceptive Oral - Biphasic	Oral
TARINA 24 FE 1 MG-20 MCG (24)/75 MG (4) TABLET	Contraceptive Oral - Monophasic	Oral
BLISOVI 24 FE 1 MG-20 MCG (24)/75 MG (4) TABLET	Contraceptive Oral - Monophasic	Oral
NORETHINDRONE 1 MG-ETHINYL ESTRADIOL 10 MCG (24)-IRON 10 MCG(2) TABLET	Contraceptive Oral - Biphasic	Oral
ASHLYNA ORAL	Contraceptive Oral - Biphasic	Oral
ISIBLOOM ORAL	Contraceptive Oral - Monophasic	Oral
ESTARYLLA ORAL	Contraceptive Oral - Monophasic	Oral
BALZIVA (28) 0.4 MG-35 MCG TABLET	Contraceptive Oral - Monophasic	Oral
NORETHINDRONE 1 MG-ETHINYL ESTRADIOL 35 MCG TABLET	Contraceptive Oral - Monophasic	Oral
NORETHINDRONE ACETATE 1.5 MG-ETHINYL ESTRADIOL 30 MCG TABLET	Contraceptive Oral - Monophasic	Oral
TRI-SPRINTEC (28) 0.18 MG(7)/0.215 MG(7)/0.25 MG(7)-35 MCG TABLET	Contraceptive Oral - Triphasic	Oral
MICROGESTIN FE 1.5/30 (28) 1.5 MG-30 MCG (21)/75 MG (7) TABLET	Contraceptive Oral - Monophasic	Oral
XULANE 150 MCG-35 MCG/24 HR TRANSDERMAL PATCH	Contraceptive Transdermal Combinations - Estrogen and Progestin Comb.	Transdermal
AUROVELA FE 1-20 (28) 1 MG-20 MCG (21)/75 MG (7) TABLET	Contraceptive Oral - Monophasic	Oral
AUROVELA 24 FE 1 MG-20 MCG (24)/75 MG (4) TABLET	Contraceptive Oral - Monophasic	Oral
KARIVA (28) 0.15 MG-0.02 MG (21)/0.01 MG (5) TABLET	Contraceptive Oral - Biphasic	Oral
CAMRESE 0.15 MG-30 MCG (84)/10 MCG(7) TABLETS,3 MONTH DOSE PACK	Contraceptive Oral - Biphasic	Oral
ERRIN 0.35 MG TABLET	Contraceptive Oral - Progestin	Oral
ZUMANDIMINE (28) 3 MG-0.03 MG TABLET	Contraceptive Oral - Monophasic	Oral
AMETHYST (28) 90 MCG-20 MCG TABLET	Contraceptive Oral - Monophasic	Oral
ESTARYLLA 0.25 MG-35 MCG TABLET	Contraceptive Oral - Monophasic	Oral
LO-ZUMANDIMINE (28) 3 MG-0.02 MG TABLET	Contraceptive Oral - Monophasic	Oral
HAILEY FE 1.5/30 (28) 1.5 MG-30 MCG (21)/75 MG (7) TABLET	Contraceptive Oral - Monophasic	Oral
VESTURA (28) 3 MG-0.02 MG TABLET	Contraceptive Oral - Monophasic	Oral
SYEDA 3 MG-0.03 MG TABLET	Contraceptive Oral - Monophasic	Oral
DROSPIRENONE (CONTRACEPTIVE) 4 MG (28) TABLET	Contraceptive Oral - Progestin	Oral
NYLIA 1/35 (28) 1 MG-35 MCG TABLET	Contraceptive Oral - Monophasic	Oral
NORETHINDRONE 1 MG-ETHINYL ESTRADIOL 20 MCG (24)-IRON 75 MG (4) TABLET	Contraceptive Oral - Monophasic	Oral
DESOGESTREL-ETHINYL ESTRADIOL 0.1 MG/0.125 MG/0.15 MG-25 MCG TABLET	Contraceptive Oral - Triphasic	Oral
JUNEL FE 24 1 MG-20 MCG (24)/75 MG (4) TABLET	Contraceptive Oral - Monophasic	Oral
LOJAIMIESS 0.1 MG-20 MCG (84)/10 MCG (7) TABLETS,3 MONTH DOSE PACK	Contraceptive Oral - Biphasic	Oral
LEVONORGESTREL 14 MCG/24 HRS (3 YRS) 13.5 MG INTRAUTERINE DEVICE	Contraceptive Intrauterine - Progesterone IUD	Intrauterine
LESSINA 0.1 MG-20 MCG TABLET	Contraceptive Oral - Monophasic	Oral
YAZ (28) 3 MG-0.02 MG TABLET	Contraceptive Oral - Monophasic	Oral
ETHYNODIOL DIACETATE-ETHINYL ESTRADIOL 1 MG-35 MCG TABLET	Contraceptive Oral - Monophasic	Oral
LOW-OGESTREL (28) 0.3 MG-30 MCG TABLET	Contraceptive Oral - Monophasic	Oral
PIRMELLA 1 MG-35 MCG TABLET	Contraceptive Oral - Monophasic	Oral
LARIN FE 1.5/30 (28) 1.5 MG-30 MCG (21)/75 MG (7) TABLET	Contraceptive Oral - Monophasic	Oral

APRI 0.15 MG-0.03 MG TABLET	Contraceptive Oral - Monophasic	Oral
ALYACEN 1/35 (28) 1 MG-35 MCG TABLET	Contraceptive Oral - Monophasic	Oral
HAILEY 24 FE 1 MG-20 MCG (24)/75 MG (4) TABLET	Contraceptive Oral - Monophasic	Oral
ESTRADIOL-DIENOGEST 3 MG/2 MG-2 MG/2 MG-3 MG/1 MG TABLET	Contraceptive Oral - Quadraphasic	Oral
SRONYX 0.1 MG-20 MCG TABLET	Contraceptive Oral - Monophasic	Oral
NORETHIN-ETHINYL ESTRADIOL-IRON 0.4 MG-35 MCG(21)/75 MG(7) CHEW TABLET	Contraceptive Oral - Monophasic	Oral
BLISOVI FE 1.5/30 (28) 1.5 MG-30 MCG (21)/75 MG (7) TABLET	Contraceptive Oral - Monophasic	Oral
NORLYDA 0.35 MG TABLET	Contraceptive Oral - Progestin	Oral
TRI-LO-MARZIA 0.18 MG/0.215 MG/0.25 MG-25 MCG TABLET	Contraceptive Oral - Triphasic	Oral
JULEBER 0.15 MG-0.03 MG TABLET	Contraceptive Oral - Monophasic	Oral
KURVELO (28) 0.15 MG-0.03 MG TABLET	Contraceptive Oral - Monophasic	Oral
VIORELE (28) 0.15 MG-0.02 MG (21)/0.01 MG (5) TABLET	Contraceptive Oral - Biphasic	Oral
ALTAVERA (28) 0.15 MG-0.03 MG TABLET	Contraceptive Oral - Monophasic	Oral
ELURYNG 0.12 MG-0.015 MG/24 HR VAGINAL RING	Contraceptives - Intravaginal, Systemic - Estrogen and Progestin Comb.	Intravaginal
NORGESTREL 0.5 MG-ETHINYL ESTRADIOL 50 MCG TABLET	Contraceptive Oral - Monophasic	Oral
TRI-LO-MILI 0.18/0.215/0.25 MG-25 MCG TABLET	Contraceptive Oral - Triphasic	Oral
ZOVIA 1-35 (28) 1 MG-35 MCG TABLET	Contraceptive Oral - Monophasic	Oral
NORTREL 1/35 (21) 1 MG-35 MCG TABLET	Contraceptive Oral - Monophasic	Oral
DEBLITANE 0.35 MG TABLET	Contraceptive Oral - Progestin	Oral
LARIN FE 1/20 (28) 1 MG-20 MCG (21)/75 MG (7) TABLET	Contraceptive Oral - Monophasic	Oral
TWIRLA 120 MCG-30 MCG/24 HR TRANSDERMAL PATCH	Contraceptive Transdermal Combinations - Estrogen and Progestin Comb.	Transdermal
DROSPIREN-E.ESTRAD-L.MEFOL 3 MG-0.02 MG-0.451 MG(24)/0.451 MG(4)TABLET	Contraceptive Oral - Monophasic	Oral
ETHYNODIOL DIACETATE-ETHINYL ESTRADIOL 1 MG-50 MCG TABLET	Contraceptive Oral - Monophasic	Oral
SPRINTEC (28) 0.25 MG-35 MCG TABLET	Contraceptive Oral - Monophasic	Oral
TRIVORA (28) 50-30 (6)/75-40(5)/125-30(10) TABLET	Contraceptive Oral - Triphasic	Oral
JUNEL FE 1/20 (28) ORAL	Contraceptive Oral - Monophasic	Oral
SEASONIQUE ORAL	Contraceptive Oral - Biphasic	Oral
VYFEMLA (28) 0.4 MG-35 MCG TABLET	Contraceptive Oral - Monophasic	Oral
PIMTREA (28) 0.15 MG-0.02 MG (21)/0.01 MG (5) TABLET	Contraceptive Oral - Biphasic	Oral
TARINA FE 1-20 EQ (28) 1 MG-20 MCG (21)/75 MG (7) TABLET	Contraceptive Oral - Monophasic	Oral
BALCOLTRA 0.1 MG-0.02 MG(21)/36.5 MG(7) TABLET	Contraceptive Oral - Monophasic	Oral
KAITLIB FE 0.8 MG-25 MCG (24)/75 MG (4) CHEWABLE TABLET	Contraceptive Oral - Monophasic	Oral
VOLNEA (28) 0.15 MG-0.02 MG (21)/0.01 MG (5) TABLET	Contraceptive Oral - Biphasic	Oral
SETLAKIN 0.15 MG-30 MCG (91) TABLETS,3 MONTH DOSE PACK	Contraceptive Oral - Monophasic	Oral
RECLIPSEN (28) 0.15 MG-0.03 MG TABLET	Contraceptive Oral - Monophasic	Oral
FALMINA (28) 0.1 MG-20 MCG TABLET	Contraceptive Oral - Monophasic	Oral
MICROGESTIN 1.5/30 (21) 1.5 MG-30 MCG TABLET	Contraceptive Oral - Monophasic	Oral
LO LOESTRIN FE ORAL	Contraceptive Oral - Biphasic	Oral
NORETHIN-ETHINYL ESTRADIOL-IRON 0.8 MG-25 MCG(24)/75 MG(4) CHEW TABLET	Contraceptive Oral - Monophasic	Oral
L.NORGEST-ETH.ESTRADIOL TRIPHASIC 50-30 (6)/75-40(5)/125-30(10) TABLET	Contraceptive Oral - Triphasic	Oral
MICROGESTIN 1/20 (21) 1 MG-20 MCG TABLET	Contraceptive Oral - Monophasic	Oral
SIMPESSE 0.15 MG-30 MCG (84)/10 MCG(7) TABLETS,3 MONTH DOSE PACK	Contraceptive Oral - Biphasic	Oral
LILLOW (28) 0.15 MG-0.03 MG TABLET	Contraceptive Oral - Monophasic	Oral
ASHLYNA 0.15 MG-30 MCG (84)/10 MCG(7) TABLETS,3 MONTH DOSE PACK	Contraceptive Oral - Biphasic	Oral
ALYACEN 7/7/7 (28) 0.5 MG/0.75 MG/1 MG-35 MCG TABLET	Contraceptive Oral - Triphasic	Oral
BLISOVI FE 1/20 (28) ORAL	Contraceptive Oral - Monophasic	Oral
MEDROXYPROGESTERONE 104 MG/0.65 ML SUBCUTANEOUS SYRINGE	Contraceptive Injectable - Progestin	Injectable
PARAGARD T 380A INTRAUTERINE	Contraceptive Intrauterine - Copper IUD	Intrauterine
MIRENA INTRAUTERINE	Contraceptive Intrauterine - Progesterone IUD	Intrauterine
LUTERA (28) ORAL	Contraceptive Oral - Monophasic	Oral
HEATHER ORAL	Contraceptive Oral - Progestin	Oral
NORETHIN-E.ESTRADIOL TRIPHASIC ORAL	Contraceptive Oral - Triphasic	Oral
DASETTA 1/35 (28) 1 MG-35 MCG TABLET	Contraceptive Oral - Monophasic	Oral
MICROGESTIN 24 FE 1 MG-20 MCG (24)/75 MG (4) TABLET	Contraceptive Oral - Monophasic	Oral
IMPLANON SUBDERMAL	Contraceptive Implant - Progestin	Implant
SKYLA INTRAUTERINE	Contraceptive Intrauterine - Progesterone IUD	Intrauterine
LEVONORGESTREL 0.1 MG-ETH.ESTRADIOL 0.02 MG(21)/IRON 36.5 MG(7) TABLET	Contraceptive Oral - Monophasic	Oral
LEVORA-28 ORAL	Contraceptive Oral - Monophasic	Oral

SLYND ORAL	Contraceptive Oral - Progestin	Oral
MARLISSA (28) ORAL	Contraceptive Oral - Monophasic	Oral
TRI-VYLIBRA ORAL	Contraceptive Oral - Triphasic	Oral
ORTHO TRI-CYCLEN LO (28) ORAL	Contraceptive Oral - Triphasic	Oral
NORTREL 7/7/7 (28) ORAL	Contraceptive Oral - Triphasic	Oral
KELNOR 1/35 (28) 1 MG-35 MCG TABLET	Contraceptive Oral - Monophasic	Oral
NORTREL 7/7/7 (28) 0.5 MG/0.75 MG/1 MG-35 MCG TABLET	Contraceptive Oral - Triphasic	Oral
CYCLAFEM 1/35 (28) 1 MG-35 MCG TABLET	Contraceptive Oral - Monophasic	Oral
PORTIA 28 0.15 MG-0.03 MG TABLET	Contraceptive Oral - Monophasic	Oral
ESTROSTEP FE-28 1-20 (5)/1-30(7)/1MG-35MCG(9) TABLET	Contraceptive Oral - Triphasic	Oral
MINASTRIN 24 FE 1 MG-20 MCG (24)/75 MG (4) CHEWABLE TABLET	Contraceptive Oral - Monophasic	Oral
HAILEY 1.5 MG-30 MCG TABLET	Contraceptive Oral - Monophasic	Oral
VYLIBRA 0.25 MG-35 MCG TABLET	Contraceptive Oral - Monophasic	Oral
BLISOVI 24 FE ORAL	Contraceptive Oral - Monophasic	Oral
NUVARING VAGINAL	Contraceptives - Intravaginal, Systemic - Estrogen and Progestin Comb.	Intravaginal
JUNEL FE 1.5/30 (28) ORAL	Contraceptive Oral - Monophasic	Oral
TRI-LO-SPRINTEC ORAL	Contraceptive Oral - Triphasic	Oral
MERZEE 1 MG-20 MCG (24)/75 MG (4) CAPSULE	Contraceptive Oral - Monophasic	Oral
TRI-ESTARYLLA (28) 0.18 MG(7)/0.215 MG(7)/0.25 MG(7)-35 MCG TABLET	Contraceptive Oral - Triphasic	Oral
LEVONORGESTREL 120 MCG-E.ESTRADIOL 30 MCG/24 HR WEEKLY TRANSDERM PATCH	Contraceptive Transdermal Combinations - Estrogen and Progestin Comb.	Transdermal
LARISSIA 0.1 MG-20 MCG TABLET	Contraceptive Oral - Monophasic	Oral
INCASSIA 0.35 MG TABLET	Contraceptive Oral - Progestin	Oral
AVIANE 0.1 MG-20 MCG TABLET	Contraceptive Oral - Monophasic	Oral
MICROGESTIN FE 1/20 (28) 1 MG-20 MCG (21)/75 MG (7) TABLET	Contraceptive Oral - Monophasic	Oral
AMETHIA 0.15 MG-30 MCG (84)/10 MCG(7) TABLETS,3 MONTH DOSE PACK	Contraceptive Oral - Biphasic	Oral
AUROVELA FE 1.5/30 (28) 1.5 MG-30 MCG (21)/75 MG (7) TABLET	Contraceptive Oral - Monophasic	Oral
SHAROBEL 0.35 MG TABLET	Contraceptive Oral - Progestin	Oral
TRI-LO-SPRINTEC 0.18 MG/0.215 MG/0.25 MG-25 MCG TABLET	Contraceptive Oral - Triphasic	Oral
OCELLA 3 MG-0.03 MG TABLET	Contraceptive Oral - Monophasic	Oral
AUROVELA 1.5/30 (21) 1.5 MG-30 MCG TABLET	Contraceptive Oral - Monophasic	Oral
LILETTA INTRAUTERINE	Contraceptive Intrauterine - Progesterone IUD	Intrauterine
JUNEL 1/20 (21) ORAL	Contraceptive Oral - Monophasic	Oral
NORA-BE ORAL	Contraceptive Oral - Progestin	Oral
NORGESTIMATE-ETHINYL ESTRADIOL ORAL	Contraceptive Oral - Monophasic	Oral
L NORGEST/EE 0.15-0.02MG/0.15-0.025MG/0.15-0.03MG/EE 0.01 MG TABS,3MO	Contraceptive Oral - Quadraphasic	Oral
LOESTRIN 1.5/30 (21) 1.5 MG-30 MCG TABLET	Contraceptive Oral - Monophasic	Oral
TILIA FE 1-20 (5)/1-30(7)/1MG-35MCG(9) TABLET	Contraceptive Oral - Triphasic	Oral
LARIN 1/20 (21) 1 MG-20 MCG TABLET	Contraceptive Oral - Monophasic	Oral
WERA (28) 0.5 MG-35 MCG TABLET	Contraceptive Oral - Monophasic	Oral
SEGESTERONE ACET 0.15 MG-ETHINYL ESTRADIOL 0.013 MG/24 HR VAGINAL RING	Contraceptives - Intravaginal, Systemic - Estrogen and Progestin Comb.	Intravaginal
LOSEASONIQUE 0.1 MG-20 MCG (84)/10 MCG (7) TABLETS,3 MONTH DOSE PACK	Contraceptive Oral - Biphasic	Oral
LOESTRIN 1/20 (21) 1 MG-20 MCG TABLET	Contraceptive Oral - Monophasic	Oral
DEPO-SUBQ PROVERA 104 104 MG/0.65 ML SUBCUTANEOUS SYRINGE	Contraceptive Injectable - Progestin	Injectable
LEVONORGESTREL IMPLANT	Contraceptive Implant - Progestin	Implant
SIMPESSE ORAL	Contraceptive Oral - Biphasic	Oral
LORYNA (28) ORAL	Contraceptive Oral - Monophasic	Oral
JOLESSA 0.15 MG-30 MCG (91) TABLETS,3 MONTH DOSE PACK	Contraceptive Oral - Monophasic	Oral
FINZALA ORAL	Contraceptive Oral - Monophasic	Oral
AUROVELA 1/20 (21) ORAL	Contraceptive Oral - Monophasic	Oral
DAYSEE ORAL	Contraceptive Oral - Biphasic	Oral
KURVELO (28) ORAL	Contraceptive Oral - Monophasic	Oral
GEMMILY ORAL	Contraceptive Oral - Monophasic	Oral
SEASONALE CONTRACEPTIVE ORAL	Contraceptive Oral - Monophasic	Oral
TRI-LEGEST FE 1-20 (5)/1-30(7)/1MG-35MCG(9) TABLET	Contraceptive Oral - Triphasic	Oral
LOESTRIN FE 1/20 (28-DAY) 1 MG-20 MCG (21)/75 MG (7) TABLET	Contraceptive Oral - Monophasic	Oral
FEMYNOR 0.25 MG-35 MCG TABLET	Contraceptive Oral - Monophasic	Oral
PREVIFEM 0.25 MG-35 MCG TABLET	Contraceptive Oral - Monophasic	Oral
JUNEL 1/20 (21) 1 MG-20 MCG TABLET	Contraceptive Oral - Monophasic	Oral
LARIN 1.5/30 (21) 1.5 MG-30 MCG TABLET	Contraceptive Oral - Monophasic	Oral

NORETHINDRONE 1 MG-ETHIN. ESTRADIOL 20 MCG (24)-IRON 75 MG (4) CAPSULE	Contraceptive Oral - Monophasic	Oral
VELIVET TRIPHASIC REGIMEN (28) 0.1 MG/0.125 MG/0.15 MG-25 MCG TABLET	Contraceptive Oral - Triphasic	Oral
VIORELE (28) ORAL	Contraceptive Oral - Biphasic	Oral
LOESTRIN 24 FE ORAL	Contraceptive Oral - Monophasic	Oral
HAILEY 24 FE ORAL	Contraceptive Oral - Monophasic	Oral
ALESSE-28 ORAL	Contraceptive Oral - Monophasic	Oral
ERRIN ORAL	Contraceptive Oral - Progestin	Oral
LEVORA-28 0.15 MG-0.03 MG TABLET	Contraceptive Oral - Monophasic	Oral
GENERESS FE 0.8 MG-25 MCG (24)/75 MG (4) CHEWABLE TABLET	Contraceptive Oral - Monophasic	Oral
BLISOVI FE 1.5/30 (28) ORAL	Contraceptive Oral - Monophasic	Oral
TRI-LINYAH ORAL	Contraceptive Oral - Triphasic	Oral
AVIANE ORAL	Contraceptive Oral - Monophasic	Oral
KAITLIB FE ORAL	Contraceptive Oral - Monophasic	Oral
ORTHO TRI-CYCLEN LO (28) 0.18 MG/0.215 MG/0.25 MG-25 MCG TABLET	Contraceptive Oral - Triphasic	Oral
ORTHO-NOVUM 1/35 (28) 1 MG-35 MCG TABLET	Contraceptive Oral - Monophasic	Oral
AZURETTE (28) 0.15 MG-0.02 MG (21)/0.01 MG (5) TABLET	Contraceptive Oral - Biphasic	Oral
DOLISHALE 90 MCG-20 MCG (28) TABLET	Contraceptive Oral - Monophasic	Oral
DROSPIREN-E.ESTRAD-L.MEFOL 3 MG-0.03 MG-0.451 MG(21)/0.451 MG(7)TABLET	Contraceptive Oral - Monophasic	Oral
LOW-OGESTREL (28) ORAL	Contraceptive Oral - Monophasic	Oral
VIENVA ORAL	Contraceptive Oral - Monophasic	Oral
NORETHINDRONE-ETHINYL ESTRADIOL TRIPHASIC 0.5/1/0.5 MG-35 MCG TABLET	Contraceptive Oral - Triphasic	Oral
GIANVI (28) 3 MG-0.02 MG TABLET	Contraceptive Oral - Monophasic	Oral
ENSKYCE ORAL	Contraceptive Oral - Monophasic	Oral
NEXTSTELLIS 3 MG-14.2 MG (28) TABLET	Contraceptive Oral - Monophasic	Oral
TRI-LO-ESTARYLLA ORAL	Contraceptive Oral - Triphasic	Oral
HAILEY ORAL	Contraceptive Oral - Monophasic	Oral
JUNEL 1.5/30 (21) ORAL	Contraceptive Oral - Monophasic	Oral
LARIN FE 1/20 (28) ORAL	Contraceptive Oral - Monophasic	Oral
LOESTRIN 1/20 (21) ORAL	Contraceptive Oral - Monophasic	Oral
NORETHINDRONE 1 MG-ETHINYL ESTRADIOL 35 MCG (21) TABLET	Contraceptive Oral - Monophasic	Oral
LYLEQ 0.35 MG TABLET	Contraceptive Oral - Progestin	Oral
MIBELAS 24 FE 1 MG-20 MCG (24)/75 MG (4) CHEWABLE TABLET	Contraceptive Oral - Monophasic	Oral
MONONESSA (28) 0.25 MG-35 MCG TABLET	Contraceptive Oral - Monophasic	Oral
DROSPIRENONE-ETHINYL ESTRADIOL ORAL	Contraceptive Oral - Monophasic	Oral
OGESTREL (28) 0.5 MG-50 MCG TABLET	Contraceptive Oral - Monophasic	Oral
TRI FEMYNOR (28) 0.18 MG(7)/0.215 MG(7)/0.25 MG(7)-35 MCG TABLET	Contraceptive Oral - Triphasic	Oral
ZOVIA 1/35E (28) 1 MG-35 MCG TABLET	Contraceptive Oral - Monophasic	Oral
ORSYTHIA 0.1 MG-20 MCG TABLET	Contraceptive Oral - Monophasic	Oral
ARANELLE (28) 0.5 MG/1 MG/0.5 MG-35 MCG TABLET	Contraceptive Oral - Triphasic	Oral
TRI-VYLIBRA (28) 0.18 MG(7)/0.215 MG(7)/0.25 MG(7)-35 MCG TABLET	Contraceptive Oral - Triphasic	Oral
ANNOVERA VAGINAL	Contraceptives - Intravaginal, Systemic - Estrogen and Progestin Comb.	Intravaginal
OCELLA ORAL	Contraceptive Oral - Monophasic	Oral
CAMRESE ORAL	Contraceptive Oral - Biphasic	Oral
MILI 0.25 MG-35 MCG TABLET	Contraceptive Oral - Monophasic	Oral
AUROVELA 1/20 (21) 1 MG-20 MCG TABLET	Contraceptive Oral - Monophasic	Oral
KELNOR 1/35 (28) ORAL	Contraceptive Oral - Monophasic	Oral
TYBLUME 0.1 MG-20 MCG CHEWABLE TABLET	Contraceptive Oral - Monophasic	Oral
JAIMIESS 0.15 MG-30 MCG (84)/10 MCG(7) TABLETS,3 MONTH DOSE PACK	Contraceptive Oral - Biphasic	Oral
NECON 0.5/35 (28) 0.5 MG-35 MCG TABLET	Contraceptive Oral - Monophasic	Oral
NORETHINDRONE (CONTRACEPTIVE) ORAL	Contraceptive Oral - Progestin	Oral
SPRINTEC (28) ORAL	Contraceptive Oral - Monophasic	Oral
LAYOLIS FE 0.8 MG-25 MCG (24)/75 MG (4) CHEWABLE TABLET	Contraceptive Oral - Monophasic	Oral
GIANVI (28) ORAL	Contraceptive Oral - Monophasic	Oral
LEVONEST (28) ORAL	Contraceptive Oral - Triphasic	Oral
ZOVIA 1-35 (28) ORAL	Contraceptive Oral - Monophasic	Oral
LOESTRIN FE 1/20 (28-DAY) ORAL	Contraceptive Oral - Monophasic	Oral
BEYAZ (28) 3 MG-0.02 MG-0.451 MG (24)/0.451 MG (4) TABLET	Contraceptive Oral - Monophasic	Oral
KELNOR 1-50 (28) 1 MG-50 MCG TABLET	Contraceptive Oral - Monophasic	Oral
MELODETTA 24 FE 1 MG-20 MCG (24)/75 MG (4) CHEWABLE TABLET	Contraceptive Oral - Monophasic	Oral

CAMRESE LO ORAL	Contraceptive Oral - Biphasic	Oral
ETONOGESTREL-ETHINYL ESTRADIOL VAGINAL	Contraceptives - Intravaginal, Systemic - Estrogen and Progestin Comb.	Intravaginal
NYLIA 1/35 (28) ORAL	Contraceptive Oral - Monophasic	Oral
DROSPIRENONE (CONTRACEPTIVE) ORAL	Contraceptive Oral - Progestin	Oral
TAYTULLA ORAL	Contraceptive Oral - Monophasic	Oral
SRONYX ORAL	Contraceptive Oral - Monophasic	Oral
VYLIBRA ORAL	Contraceptive Oral - Monophasic	Oral
SYEDA ORAL	Contraceptive Oral - Monophasic	Oral
NECON 7/7/7 (28) ORAL	Contraceptive Oral - Triphasic	Oral
LARISSIA ORAL	Contraceptive Oral - Monophasic	Oral
AUBRA 0.1 MG-20 MCG TABLET	Contraceptive Oral - Monophasic	Oral
ESTRADIOL VALERATE-DIENOGEST ORAL	Contraceptive Oral - Quadraphasic	Oral
NIKKI (28) ORAL	Contraceptive Oral - Monophasic	Oral
TRINESSA (28) ORAL	Contraceptive Oral - Triphasic	Oral
DESOGEN 0.15 MG-0.03 MG TABLET	Contraceptive Oral - Monophasic	Oral
PIRMELLA 0.5/0.75/1 MG-35 MCG TABLET	Contraceptive Oral - Triphasic	Oral
ORSYTHIA ORAL	Contraceptive Oral - Monophasic	Oral
MIBELAS 24 FE ORAL	Contraceptive Oral - Monophasic	Oral
JASMIEL (28) 3 MG-0.02 MG TABLET	Contraceptive Oral - Monophasic	Oral
TAYTULLA 1 MG-20 MCG (24)/75 MG (4) CAPSULE	Contraceptive Oral - Monophasic	Oral
MICROGESTIN 24 FE ORAL	Contraceptive Oral - Monophasic	Oral
ZENCHENT (28) 0.4 MG-35 MCG TABLET	Contraceptive Oral - Monophasic	Oral
NOR-Q-D ORAL	Contraceptive Oral - Progestin	Oral
LEVONORGESTREL-ETHINYL ESTRAD ORAL	Contraceptive Oral - Monophasic	Oral
LESSINA ORAL	Contraceptive Oral - Monophasic	Oral
ORTHO TRI-CYCLEN (28) ORAL	Contraceptive Oral - Triphasic	Oral
LEVLEN (28) ORAL	Contraceptive Oral - Monophasic	Oral
LARIN 24 FE ORAL	Contraceptive Oral - Monophasic	Oral
CAMRESE LO 0.1 MG-20 MCG (84)/10 MCG (7) TABLETS,3 MONTH DOSE PACK	Contraceptive Oral - Biphasic	Oral
LEVONORGESTREL INTRAUTERINE	Contraceptive Intrauterine - Progesterone IUD	Intrauterine
JUNEL FE 24 ORAL	Contraceptive Oral - Monophasic	Oral
ORTHO-NOVUM 1/35 (28) ORAL	Contraceptive Oral - Monophasic	Oral
CAMILA 0.35 MG TABLET	Contraceptive Oral - Progestin	Oral
MICROGESTIN 1/20 (21) ORAL	Contraceptive Oral - Monophasic	Oral
FALMINA (28) ORAL	Contraceptive Oral - Monophasic	Oral
MONO-LINYAH ORAL	Contraceptive Oral - Monophasic	Oral
LEVONEST (28) 50-30 (6)/75-40(5)/125-30(10) TABLET	Contraceptive Oral - Triphasic	Oral
TARINA FE 1/20 (28) ORAL	Contraceptive Oral - Monophasic	Oral
YASMIN (28) ORAL	Contraceptive Oral - Monophasic	Oral
MIRCETTE (28) 0.15 MG-0.02 MG (21)/0.01 MG (5) TABLET	Contraceptive Oral - Biphasic	Oral
NORDETTE (28) ORAL	Contraceptive Oral - Monophasic	Oral
ORTHO-CYCLEN (28) ORAL	Contraceptive Oral - Monophasic	Oral
MONONESSA (28) ORAL	Contraceptive Oral - Monophasic	Oral
LEVONORGESTREL 0.1 MG-ETHINYL ESTRADIOL 20 MCG CHEWABLE TABLET	Contraceptive Oral - Monophasic	Oral
AUROVELA FE 1-20 (28) ORAL	Contraceptive Oral - Monophasic	Oral
MICROGESTIN 1.5/30 (21) ORAL	Contraceptive Oral - Monophasic	Oral
XULANE TD	Contraceptive Transdermal Combinations - Estrogen and Progestin Comb.	Transdermal
TRI-LO-MARZIA ORAL	Contraceptive Oral - Triphasic	Oral
LEVORA 0.15/30 (28) ORAL	Contraceptive Oral - Monophasic	Oral
ORTHO-NOVUM 1/50 (28) ORAL	Contraceptive Oral - Monophasic	Oral
NORLYDA ORAL	Contraceptive Oral - Progestin	Oral
INTROVALE 0.15 MG-30 MCG (91) TABLETS,3 MONTH DOSE PACK	Contraceptive Oral - Monophasic	Oral
AUBRA ORAL	Contraceptive Oral - Monophasic	Oral
NORTREL 0.5/35 (28) ORAL	Contraceptive Oral - Monophasic	Oral
TRI-PREVIFEM (28) 0.18 MG(7)/0.215 MG(7)/0.25 MG(7)-35 MCG TABLET	Contraceptive Oral - Triphasic	Oral
LEVONORG-ETH ESTRAD TRIPHASIC ORAL	Contraceptives Oral - Triphasic	Oral
TRI-SPRINTEC (28) ORAL	Contraceptive Oral - Triphasic	Oral
FINZALA 1 MG-20 MCG (24)/75 MG (4) CHEWABLE TABLET	Contraceptive Oral - Monophasic	Oral
TRIPHASIL-28 ORAL	Contraceptive Oral - Triphasic	Oral
CHARLOTTE 24 FE 1 MG-20 MCG (24)/75 MG (4) CHEWABLE TABLET	Contraceptive Oral - Monophasic	Oral
COPPER INTRAUTERINE	Contraceptive Intrauterine - Copper IUD	Intrauterine
AMETHIA ORAL	Contraceptive Oral - Biphasic	Oral

LARIN FE 1.5/30 (28) ORAL	Contraceptive Oral - Monophasic	Oral
PHILITH 0.4 MG-35 MCG TABLET	Contraceptive Oral - Monophasic	Oral
NECON 1/35 (28) ORAL	Contraceptive Oral - Monophasic	Oral
CRYSELLE (28) ORAL	Contraceptive Oral - Monophasic	Oral
MINASTRIN 24 FE ORAL	Contraceptive Oral - Monophasic	Oral
AUBRA EQ ORAL	Contraceptive Oral - Monophasic	Oral
DROSPIRENONE-ESTETROL ORAL	Contraceptive Oral - Monophasic	Oral
PIRMELLA ORAL	Contraceptive Oral - Monophasic	Oral
ENPRESSE 50-30 (6)/75-40(5)/125-30(10) TABLET	Contraceptive Oral - Triphasic	Oral
ORTHO MICRONOR 0.35 MG TABLET	Contraceptive Oral - Progestin	Oral
DESOGESTREL-ETHINYL ESTRADIOL ORAL	Contraceptive Oral - Monophasic	Oral
AUROVELA 24 FE ORAL	Contraceptive Oral - Monophasic	Oral
CHATEAL (28) ORAL	Contraceptive Oral - Monophasic	Oral
JAIMIESS ORAL	Contraceptive Oral - Biphasic	Oral
AMETHIA LO ORAL	Contraceptive Oral - Biphasic	Oral
NORGESTREL ORAL	Contraceptive Oral - Progestin	Oral
AUBRA EQ 0.1 MG-20 MCG TABLET	Contraceptive Oral - Monophasic	Oral
MICROGESTIN FE 1.5/30 (28) ORAL	Contraceptive Oral - Monophasic	Oral
MICROGESTIN FE 1/20 (28) ORAL	Contraceptive Oral - Monophasic	Oral
TRINESSA LO 0.18 MG/0.215 MG/0.25 MG-25 MCG TABLET	Contraceptive Oral - Triphasic	Oral
ALTAVERA (28) ORAL	Contraceptive Oral - Monophasic	Oral
TRIVORA (28) ORAL	Contraceptive Oral - Triphasic	Oral
ZOVIA 1/35E (28) ORAL	Contraceptive Oral - Monophasic	Oral
NORETHINDRONE-E.ESTRADIOL-IRON ORAL	Contraceptive Oral - Monophasic	Oral
DASETTA 7/7/7 (28) 0.5 MG(7)/0.75 MG(7)/1 MG(7)-35 MCG TABLET	Contraceptive Oral - Triphasic	Oral
KARIVA (28) ORAL	Contraceptive Oral - Biphasic	Oral
FEMYNOR ORAL	Contraceptive Oral - Monophasic	Oral
LO MINASTRIN FE ORAL	Contraceptive Oral - Monophasic	Oral
DEPO-PROVERA 150 MG/ML INTRAMUSCULAR SUSPENSION	Contraceptive Injectable - Progestin	Injectable
HAILEY FE 1.5/30 (28) ORAL	Contraceptive Oral - Monophasic	Oral
LEVONORGEST-ETH.ESTRADIOL-IRON ORAL	Contraceptive Oral - Monophasic	Oral
LARIN 1/20 (21) ORAL	Contraceptive Oral - Monophasic	Oral
LEVORA 0.15/30 (28) 0.15 MG-0.03 MG TABLET	Contraceptive Oral - Monophasic	Oral
EMOQUETTE 0.15 MG-0.03 MG TABLET	Contraceptive Oral - Monophasic	Oral
CYCLAFEM 1/35 (28) ORAL	Contraceptive Oral - Monophasic	Oral
ORTHO-CYCLEN (28) 0.25 MG-35 MCG TABLET	Contraceptive Oral - Monophasic	Oral
ORTHO EVRA 150 MCG-35 MCG/24 HR TRANSDERMAL PATCH	Contraceptive Transdermal Combinations - Estrogen and Progestin Comb.	Transdermal
SEASONIQUE 0.15 MG-30 MCG (84)/10 MCG(7) TABLETS,3 MONTH DOSE PACK	Contraceptive Oral - Biphasic	Oral
CAZIANT (28) 0.1 MG/0.125 MG/0.15 MG-25 MCG TABLET	Contraceptive Oral - Triphasic	Oral
QUASENSE 0.15 MG-30 MCG (91) TABLETS,3 MONTH DOSE PACK	Contraceptive Oral - Monophasic	Oral
LOESTRIN FE 1.5/30 (28-DAY) 1.5 MG-30 MCG (21)/75 MG (7) TABLET	Contraceptive Oral - Monophasic	Oral
DROSPIRENONE-E.ESTRADIOL-LM.FA ORAL	Contraceptive Oral - Monophasic	Oral
NORETHIN-ETH ESTRAD BIPHASIC ORAL	Contraceptive Oral - Biphasic	Oral
TULANA ORAL	Contraceptive Oral - Progestin	Oral
BALZIVA (28) ORAL	Contraceptive Oral - Monophasic	Oral
APRI ORAL	Contraceptive Oral - Monophasic	Oral
MELODETTA 24 FE ORAL	Contraceptive Oral - Monophasic	Oral
SAFYRAL 3 MG-0.03 MG-0.451 MG (21)/0.451 MG (7) TABLET	Contraceptive Oral - Monophasic	Oral
WYMZYA FE 0.4 MG-35 MCG (21)/75 MG (7) CHEWABLE TABLET	Contraceptive Oral - Monophasic	Oral
TULANA 0.35 MG TABLET	Contraceptive Oral - Progestin	Oral
TRINESSA (28) 0.18 MG(7)/0.215 MG(7)/0.25 MG(7)-35 MCG TABLET	Contraceptive Oral - Triphasic	Oral
AUROVELA FE 1.5/30 (28) ORAL	Contraceptive Oral - Monophasic	Oral
LO-OVRAL (8) ORAL	Contraceptive Oral - Monophasic	Oral
LOMEDIA 24 FE 1 MG-20 MCG (24)/75 MG (4) TABLET	Contraceptive Oral - Monophasic	Oral
JOLIVETTE 0.35 MG TABLET	Contraceptive Oral - Progestin	Oral
NORTREL 1/35 (28) ORAL	Contraceptive Oral - Monophasic	Oral
TRI FEMYNOR ORAL	Contraceptive Oral - Triphasic	Oral
MYZILRA ORAL	Contraceptive Oral - Triphasic	Oral
CAMILA ORAL	Contraceptive Oral - Progestin	Oral
NECON 7/7/7 (28) 0.5 MG/0.75 MG/1 MG-35 MCG TABLET	Contraceptive Oral - Triphasic	Oral
AMETHIA LO 0.1 MG-20 MCG (84)/10 MCG (7) TABLETS,3 MONTH DOSE PACK	Contraceptive Oral - Biphasic	Oral
DEPO-SUBQ PROVERA 104 SUBCUT	Contraceptive Injectable - Progestin	Injectable
PORTIA 28 ORAL	Contraceptive Oral - Monophasic	Oral

ETONOGESTREL SUBDERMAL	Contraceptive Implant - Progestin	Implant
LYZA ORAL	Contraceptive Oral - Progestin	Oral
CYRED EQ 0.15 MG-0.03 MG TABLET	Contraceptive Oral - Monophasic	Oral
VELIVET TRIPHASIC REGIMEN (28) ORAL	Contraceptive Oral - Triphasic	Oral
MICRONOR (28) ORAL	Contraceptive Oral - Progestin	Oral
ORTHO MICRONOR ORAL	Contraceptive Oral - Progestin	Oral
TRINESSA LO ORAL	Contraceptive Oral - Triphasic	Oral
NORETHINDRONE-ETHIN ESTRADIOL ORAL	Contraceptive Oral - Monophasic	Oral
ORTHO TRI-CYCLEN (28) 0.18 MG(7)/0.215 MG(7)/0.25 MG(7)-35 MCG TABLET	Contraceptive Oral - Triphasic	Oral
CHATEAL EQ (28) 0.15 MG-0.03 MG TABLET	Contraceptive Oral - Monophasic	Oral
TRI-PREVIFEM (28) ORAL	Contraceptive Oral - Triphasic	Oral
FEMCON FE ORAL	Contraceptive Oral - Monophasic	Oral
GILDESS FE 1/20 (28) ORAL	Contraceptive Oral - Monophasic	Oral
NORGESTREL-ETHINYL ESTRADIOL ORAL	Contraceptive Oral - Monophasic	Oral
ORTHO-NOVUM 7/7/7 (28) 0.5 MG/0.75 MG/1 MG-35 MCG TABLET	Contraceptive Oral - Triphasic	Oral
OGESTREL (28) ORAL	Contraceptive Oral - Monophasic	Oral
NATAZIA ORAL	Contraceptive Oral - Quadraphasic	Oral
NORTREL 1/35 (21) ORAL	Contraceptive Oral - Monophasic	Oral
BEYAZ ORAL	Contraceptive Oral - Monophasic	Oral
NECON 1/35 (28) 1 MG-35 MCG TABLET	Contraceptive Oral - Monophasic	Oral
GILDESS 1/20 (21) 1 MG-20 MCG TABLET	Contraceptive Oral - Monophasic	Oral
ZARAH 3 MG-0.03 MG TABLET	Contraceptive Oral - Monophasic	Oral
NORETHINDRONE 1 MG-MESTRANOL 50 MCG TABLET	Contraceptive Oral - Monophasic	Oral
NOR-QD ORAL	Contraceptive Oral - Progestin	Oral
VYFEMLA (28) ORAL	Contraceptive Oral - Monophasic	Oral
JOLIVETTE ORAL	Contraceptive Oral - Progestin	Oral
GILDESS FE ORAL	Contraceptive Oral - Monophasic	Oral
ZARAH ORAL	Contraceptive Oral - Monophasic	Oral
GILDESS FE 1/20 (28) 1 MG-20 MCG (21)/75 MG (7) TABLET	Contraceptive Oral - Monophasic	Oral
LOMEDIA 24 FE ORAL	Contraceptive Oral - Monophasic	Oral
LO-OVRAL (21) ORAL	Contraceptive Oral - Monophasic	Oral
MYZILRA 50-30 (6)/75-40(5)/125-30(10) TABLET	Contraceptive Oral - Triphasic	Oral
ZOVIA 1/50E (28) 1 MG-50 MCG TABLET	Contraceptive Oral - Monophasic	Oral
GILDESS 1.5/30 (21) 1.5 MG-30 MCG TABLET	Contraceptive Oral - Monophasic	Oral
GILDESS FE 1.5/30 (28) 1.5 MG-30 MCG (21)/75 MG (7) TABLET	Contraceptive Oral - Monophasic	Oral
ZENCHENT FE 0.4 MG-35 MCG (21)/75 MG (7) CHEWABLE TABLET	Contraceptive Oral - Monophasic	Oral
QUASENSE ORAL	Contraceptive Oral - Monophasic	Oral
GENERESS FE ORAL	Contraceptive Oral - Monophasic	Oral
TRI-ESTARYLLA ORAL	Contraceptive Oral - Triphasic	Oral
NORETHIN-ETH ESTRAD BIPHASIC 0.5 MG-35 MCG(10)/1 MG-35 MCG(11) TABLET	Contraceptive Oral - Biphasic	Oral
CYCLAFEM 7/7/7 (28) 0.5 MG/0.75 MG/1 MG-35 MCG TABLET	Contraceptive Oral - Triphasic	Oral
ZENCHENT FE ORAL	Contraceptive Oral - Monophasic	Oral
PORTIA ORAL	Contraceptive Oral - Monophasic	Oral
NOR-Q-D 0.35 MG TABLET	Contraceptive Oral - Progestin	Oral
AZURETTE (28) ORAL	Contraceptive Oral - Biphasic	Oral
VESTURA (28) ORAL	Contraceptive Oral - Monophasic	Oral
RECLIPSEN (28) ORAL	Contraceptive Oral - Monophasic	Oral
MARLISSA ORAL	Contraceptive Oral - Monophasic	Oral
KURVELO ORAL	Contraceptive Oral - Monophasic	Oral
FEMCON FE 0.4 MG-35 MCG (21)/75 MG (7) CHEWABLE TABLET	Contraceptive Oral - Monophasic	Oral
NECON 1/50 (28) 1 MG-50 MCG TABLET	Contraceptive Oral - Monophasic	Oral
HAILEY FE 1/20 (28) ORAL	Contraceptive Oral - Monophasic	Oral
ESTROSTEP FE-28 ORAL	Contraceptive Oral - Triphasic	Oral
ARANELLE (28) ORAL	Contraceptive Oral - Triphasic	Oral
LEENA 28 0.5 MG/1 MG/0.5 MG-35 MCG TABLET	Contraceptive Oral - Triphasic	Oral
MIRCETTE (28) ORAL	Contraceptive Oral - Biphasic	Oral
LAYOLIS FE ORAL	Contraceptive Oral - Monophasic	Oral
NORETHINDRONE-ETHINYL ESTRADIOL 1 MG-50 MCG TABLET	Contraceptive Oral - Monophasic	Oral
NORDETTE ORAL	*Unspecified	
DESOGEN ORAL	Contraceptive Oral - Monophasic	Oral
LOESTRIN 1.5/30 (21) ORAL	Contraceptive Oral - Monophasic	Oral
GILDESS 1.5/30 (21) ORAL	Contraceptive Oral - Monophasic	Oral
EMOQUETTE ORAL	Contraceptive Oral - Monophasic	Oral
GILDESS 24 FE ORAL	Contraceptive Oral - Monophasic	Oral

NORETHINDRONE 1 MG-E.ESTRADIOL 10 MCG(24)-IRON 10 MG(2)CHEW TAB,TABLET	Contraceptive Oral - Monophasic	Oral
INTROVALE ORAL	Contraceptive Oral - Monophasic	Oral
WERA (28) ORAL	Contraceptive Oral - Monophasic	Oral
MEDROXYPROGESTERONE SUBCUT	Contraceptive Injectable - Progestin	Injectable
LILLOW ORAL	Contraceptive Oral - Monophasic	Oral
NORETH-ETHINYL ESTRADIOL-IRON ORAL	Contraceptive Oral - Monophasic	Oral
RIVELSA ORAL	Contraceptive Oral - Quadraphasic	Oral
QUARTETTE ORAL	Contraceptive Oral - Quadraphasic	Oral
DESOG-E.ESTRADIOL/E.ESTRADIOL ORAL	Contraceptive Oral - Biphasic	Oral
GILDESS 1/20 (21) ORAL	Contraceptive Oral - Monophasic	Oral
ORTHO-NOVUM 7/7/7 (28) ORAL	Contraceptive Oral - Triphasic	Oral
LYZA 0.35 MG TABLET	Contraceptive Oral - Progestin	Oral
ELINEST ORAL	Contraceptive Oral - Monophasic	Oral
PREVIFEM ORAL	Contraceptive Oral - Monophasic	Oral
JULEBER ORAL	Contraceptive Oral - Monophasic	Oral
DASETTA 1/35 (28) ORAL	Contraceptive Oral - Monophasic	Oral
SAFYRAL ORAL	Contraceptive Oral - Monophasic	Oral
AMETHYST ORAL	Contraceptive Oral - Monophasic	Oral
DASETTA 7/7/7 (28) ORAL	Contraceptive Oral - Triphasic	Oral
ZENCHENT (28) ORAL	Contraceptive Oral - Monophasic	Oral
CHATEAL ORAL	Contraceptive Oral - Monophasic	Oral
JOLESSA ORAL	Contraceptive Oral - Monophasic	Oral
CAZIANT (28) ORAL	Contraceptive Oral - Triphasic	Oral
WYMZYA FE ORAL	Contraceptive Oral - Monophasic	Oral
LO-OVRAL (28) ORAL	Contraceptive Oral - Monophasic	Oral
CYCLAFEM 7/7/7 (28) ORAL	Contraceptive Oral - Triphasic	Oral
LEENA 28 ORAL	Contraceptive Oral - Triphasic	Oral
GILDESS FE 1.5/30 (28) ORAL	Contraceptive Oral - Monophasic	Oral
GILDESS ORAL	Contraceptive Oral - Monophasic	Oral
LARIN 1.5/30 (21) ORAL	Contraceptive Oral - Monophasic	Oral
PIMTREA (28) ORAL	Contraceptive Oral - Biphasic	Oral
QUARTETTE 0.15 MG-20 MCG/0.15 MG-25 MCG TABLETS,3 MONTH DOSE PACK	Contraceptive Oral - Quadraphasic	Oral
NORETHINDRONE-MESTRANOL ORAL	Contraceptive Oral - Monophasic	Oral
ORTHO EVRA TD	Contraceptive Transdermal Combinations - Estrogen and Progestin Comb.	Transdermal
ENPRESSE ORAL	Contraceptive Oral - Triphasic	Oral
SETLAKIN ORAL	Contraceptive Oral - Monophasic	Oral
RAJANI (28) 3 MG-0.02 MG-0.451 MG (24)/0.451 MG (4) TABLET	Contraceptive Oral - Monophasic	Oral
BEKYREE (28) ORAL	Contraceptive Oral - Biphasic	Oral
PHILITH ORAL	Contraceptive Oral - Monophasic	Oral
TRI-LEGEST FE ORAL	Contraceptive Oral - Triphasic	Oral
LOESTRIN 24 FE 1 MG-20 MCG (24)/75 MG (4) TABLET	Contraceptive Oral - Monophasic	Oral
INTRAUTERINE DEVICE (IUD)	Medical Supplies and DME - IUDs	Intrauterine
BRIELLYN 0.4 MG-35 MCG TABLET	Contraceptive Oral - Monophasic	Oral
ORTHO-CEPT (28) ORAL	Contraceptive Oral - Monophasic	Oral
KIMIDESS (28) 0.15 MG-0.02 MG (21)/0.01 MG (5) TABLET	Contraceptive Oral - Biphasic	Oral
GIANVI ORAL	Contraceptive Oral - Monophasic	Oral
AZURETTE ORAL	Contraceptive Oral - Biphasic	Oral
LOESTRIN FE 1/20 (28) ORAL	Contraceptive Oral - Monophasic	Oral
SHAROBEL ORAL	Contraceptive Oral - Progestin	Oral
CHATEAL (28) 0.15 MG-0.03 MG TABLET	Contraceptive Oral - Monophasic	Oral
ORTHO-CEPT (28) 0.15 MG-0.03 MG TABLET	Contraceptive Oral - Monophasic	Oral
ETHYNODIOL DIAC-ETH ESTRADIOL ORAL	Contraceptive Oral - Monophasic	Oral
YAZ 28 ORAL	Contraceptive Oral - Monophasic	Oral
TARINA FE ORAL	Contraceptive Oral - Monophasic	Oral
ORTHO TRI-CYCLEN LO ORAL	Contraceptive Oral - Triphasic	Oral
YASMIN 28 ORAL	Contraceptive Oral - Monophasic	Oral
ALESSE (28) 0.1 MG-20 MCG TABLET	Contraceptive Oral - Monophasic	Oral
NECON 0.5/35 (28) ORAL	Contraceptive Oral - Monophasic	Oral
OVCON-35 (28) ORAL	Contraceptive Oral - Monophasic	Oral
CYRED 0.15 MG-0.03 MG TABLET	Contraceptive Oral - Monophasic	Oral
OVCON-50 (28) ORAL	Contraceptive Oral - Monophasic	Oral
NORINYL 1+35 (28) ORAL	Contraceptive Oral - Monophasic	Oral
PROGESTERONE INTRAUTERINE	Contraceptive Intrauterine - Progesterone IUD	Intrauterine

KARIVA ORAL	Contraceptive Oral - Biphasic	Oral
LORYNA ORAL	Contraceptive Oral - Monophasic	Oral
LOSEASONIQUE ORAL	Contraceptive Oral - Biphasic	Oral
SEASONALE ORAL	Contraceptive Oral - Monophasic	Oral
LOESTRIN FE 1.5/30 (28) ORAL	Contraceptive Oral - Monophasic	Oral
MIRCETTE ORAL	Contraceptive Oral - Biphasic	Oral
DEMULEN 1/50 (21) ORAL	Contraceptive Oral - Monophasic	Oral
LO-OVRAL (28) 0.3 MG-30 MCG TABLET	Contraceptive Oral - Monophasic	Oral
DEPO-PROVERA 150 MG/ML INTRAMUSCULAR SYRINGE	Contraceptive Injectable - Progestin	Injectable
VESTURA ORAL	Contraceptive Oral - Monophasic	Oral
CAZIANT ORAL	Contraceptive Oral - Triphasic	Oral
ORTHO-NOVUM 7/7/7 (21) ORAL	Contraceptive Oral - Triphasic	Oral
TRI-LEVLEN (28) ORAL	Contraceptive Oral - Triphasic	Oral
MODICON (28) 0.5 MG-35 MCG TABLET	Contraceptive Oral - Monophasic	Oral
TILIA FE ORAL	Contraceptive Oral - Triphasic	Oral
SEASONALE (91) 0.15 MG-30 MCG TABLETS,3 MONTH DOSE PACK	Contraceptive Oral - Monophasic	Oral
DEMULEN 1/50 (28) ORAL	Contraceptive Oral - Monophasic	Oral
NORGESTIMATE-ETHINYL ESTRADIOL 0.18 MG/0.215MG/0.25MG-35 MCG(21)TABLET	Contraceptive Oral - Triphasic	Oral
NORDETTE-28 ORAL	Contraceptive Oral - Monophasic	Oral
NORINYL 1/35 (28) 1 MG-35 MCG TABLET	Contraceptive Oral - Monophasic	Oral
NECON 1/50 (28) ORAL	Contraceptive Oral - Monophasic	Oral
CYCLESSA ORAL	Contraceptive Oral - Triphasic	Oral
NORDETTE (28) 0.15 MG-0.03 MG TABLET	Contraceptive Oral - Monophasic	Oral
NORETHINDRONE-ETHINYL ESTRADIOL BIPHASIC 0.5-35/1-35 MG-MCG (7) TABLET	Contraceptive Oral - Biphasic	Oral
LYBREL ORAL	Contraceptive Oral - Monophasic	Oral
ZEOSA ORAL	Contraceptive Oral - Monophasic	Oral
L NORGEST&E ESTRADIOL-E ESTRAD ORAL	Contraceptive Oral - Biphasic	Oral
DEMULEN 1/50 (28) 1 MG-50 MCG TABLET	Contraceptive Oral - Monophasic	Oral
GEMMILY 1 MG-20 MCG (24)/75 MG (4) CAPSULE	Contraceptive Oral - Monophasic	Oral
VELIVET ORAL	Contraceptive Oral - Triphasic	Oral
BREVICON (28) ORAL	Contraceptive Oral - Monophasic	Oral
NYLIA 7/7/7 (28) 0.5/0.75/1 MG-35 MCG TABLET	Contraceptive Oral - Triphasic	Oral
CYCLESSA (28) ORAL	Contraceptive Oral - Triphasic	Oral
TRI-NORINYL (28) ORAL	Contraceptive Oral - Triphasic	Oral
NORGESTREL 0.075 MG TABLET	Contraceptive Oral - Progestin	Oral
mirena		Intrauterine
intrauterine		Intrauterine
copper		Intrauterine
lng		Intrauterine
i.u.d.		Intrauterine
iud		Intrauterine
-t		Intrauterine
implant		Implant
subdermal		Implant
injection		Injectable
provera		Injectable
intramuscular		Injectable
depo		Injectable
medroxyprogesterone acetate		Injectable
dmpa		Injectable
subcut		Injectable
ring		Intravaginal
nuvaring		Intravaginal
intravaginal		Intravaginal
transderm		Transdermal
td		Transdermal
evra		Transdermal
patch		Transdermal
oral		Oral
ocp		Oral
pill		Oral
tablet		Oral
ocs		Oral
tabs		Oral

capsule		Oral
tri		Oral
micronor		Oral
loestrin		Oral
nordette		Oral
ortho		Oral
bcp		Oral
pop		Oral
apri		Oral
norethindrone		Oral
seasonal		Oral
lutera		Oral
estradiol		Oral
demulen		Oral
drospirenone-ethinyl		Oral
norgestimate-ethinyl estradiol		Oral
levonorgestrel-ethinyl estradiol		Oral
norgestrel-ethinyl estradiol		Oral
estrostep		Oral
lo		Oral

Supplementary Table 3: Prompts and system messages.
Prompts and systems messages used to identify contraceptives started, stopped, or reasons for stopping from clinical notes. Six different approaches, consisting of 3 system messages each combined with 2 prompts, were used to extract information. Prompts were provided to the language model following the clinical note text.

**System message**	**None**	<Blank>
	**General**	You are an expert physician extracting information from clinical notes.
	**Specialist**	You are an expert women's health researcher extracting information from clinical notes.
**Prompt**	**Default**	Task: Contraceptives can be classified into "Injectable", "Intravaginal", "Transdermal", "Intrauterine", "Oral", or "Implant". Answer the following questions -<br>1. What new contraceptive was suggested, proposed, or prescribed? If the patient is not starting a new contraceptive, write "NA"<br>2. What was the previous contraceptive the patient used? If none, write "NA"<br>3. In 20 words or less, why was the last contraceptive stopped or planned to be stopped? If the contraceptive was not stopped, write "NA"<br>Use the following format: {"new_contraceptive":str, "last_contraceptive":str, "Reason contraceptive stopped":str}<br>Answer:
	**Dictionary**	Task: Contraceptives can be classified into "Injectable", "Intravaginal", "Transdermal", "Intrauterine", "Oral", or "Implant". Extract this information from the clinical note about contraceptives in JSON format:<br>{<br>"new_contraceptive": {"Type":str, "Description":"What new contraceptive was suggested, proposed, or prescribed? If the patient is not starting a new contraceptive, write "NA""},<br>"last_contraceptive":{"Type":str,"Description":"What was the last contraceptive the patient used? If none, write "NA""},<br>"Reason contraceptive stopped":{"Type":str, "Description":"In 20 words or less, why was the last contraceptive stopped or planned to be stopped?<br>If the contraceptive was not stopped, write "NA""}<br>}<br>Answer:

Supplementary Table 4: Proportion of patients switching between different classes of contraceptives
Proportion of each contraceptive switch identified using structured medication prescription data.

Previous contraceptive	New contraceptive	Count	Proportion
Oral	Intravaginal	205	10.44%
Intravaginal	Oral	172	8.76%
Oral	Intrauterine	165	8.40%
Oral	Implant	149	7.59%
Injectable	Oral	141	7.18%
Oral	Injectable	141	7.18%
Oral	Transdermal	131	6.67%
Implant	Oral	122	6.21%
Transdermal	Oral	114	5.80%
Injectable	Implant	68	3.46%
Intrauterine	Oral	62	3.16%
Intravaginal	Intrauterine	59	3.00%
Transdermal	Injectable	50	2.55%
Transdermal	Implant	42	2.14%
Implant	Intravaginal	35	1.78%
Transdermal	Intravaginal	30	1.53%
Intravaginal	Implant	29	1.48%
Implant	Transdermal	29	1.48%
Intravaginal	Transdermal	28	1.43%
Injectable	Transdermal	28	1.43%
Implant	Injectable	27	1.37%
Injectable	Intravaginal	26	1.32%
Intravaginal	Injectable	20	1.02%
Intrauterine	Intravaginal	20	1.02%
Transdermal	Intrauterine	16	0.81%
Implant	Intrauterine	15	0.76%
Intrauterine	Transdermal	12	0.61%
Injectable	Intrauterine	12	0.61%
Intrauterine	Implant	10	0.51%
Intrauterine	Injectable	6	0.31%

Supplementary Table 5: Reasons for contraceptive switching in a subset of manually labeled clinical notes.
Clinical annotators provided the class of contraceptive started, stopped, and whether the reason for contraceptive switching extracted using GPT4 was correct for a subset of 93 clinical notes. Additional information, including whether the label was redacted or inferred was also included by the clinical annotator. Corresponding information from structured medication prescription data is also included to demonstrate discordance between information documented in clinical notes and medication prescription information.

ID	Contraceptive started - Human label	Contraceptive stopped - Human label	GPT4 Extracted Reason	Reason correct?	Hallucination in reason?	Start label inferred by evaluator?	Stop label inferred by evaluator?	Start label redacted?	Stop label redacted?	Contraceptive started - Structured data	Contraceptive stopped - Structured data
0	Intrauterine	None	None	Y	N	0	0	0	0	Intrauterine	Intravaginal
1	Intrauterine	None	None	Y	N	0	0	0	0	Intrauterine	Intravaginal
2	None	None	None	Y	N	0	0	0	0	Transdermal	Intrauterine
3	Intravaginal	None	None	Y	N	0	0	0	0	Intravaginal	Transdermal
4	Intravaginal	Intrauterine	Continued irregular intermenstrual bleeding for 1 year	Y	N	1	0	0	0	Intravaginal	Intrauterine
5	Intravaginal	None	None	Y	N	0	0	0	0	Intravaginal	Oral
6	None	Injectable	Continuous menstrual bleeding after administration	Y	N	0	1	0	0	Oral	Injectable
7	None	Implant	Patient was worried about weight gain with the contraceptive method.	Y	N	0	1	1	0	Oral	Implant
8	Intrauterine	None	None	Y	N	0	0	0	0	Intrauterine	Transdermal
9	None	None	None	Y	N	0	0	0	0	Oral	Injectable
10	None	None	None	Y	N	0	0	0	0	Transdermal	Oral
11	None	Oral	Discontinued due to mood symptoms	Y	N	0	0	0	0	Transdermal	Oral
12	Implant	Oral	None	Y	N	1	1	0	0	Implant	Oral
13	Oral	Intravaginal	Hard for her to remember	Y	N	0	0	0	0	Oral	Intravaginal
14	Oral	None	None	Y	N	0	0	0	0	Oral	Transdermal
15	Implant	None	None	Y	N	0	0	0	0	Implant	Oral
16	Intravaginal	Oral	Patient has problems with compliance	Y	N	1	1	0	0	Intravaginal	Oral
17	Oral	None	None	Y	N	0	0	0	0	Oral	Intravaginal
18	None	Oral	Patient often forgets to use OCPs	Y	N	0	0	0	0	Injectable	Oral
19	None	Intrauterine	None	Y	N	0	0	0	0	Transdermal	Injectable
20	Intrauterine	None	None	Y	N	0	0	0	0	Intrauterine	Transdermal
21	Intrauterine	None	None	Y	N	0	0	0	0	Intrauterine	Oral
22	Oral	Intravaginal	Caused severe depression and suicidal ideation	Y	N	0	0	0	0	Oral	Intravaginal
23	Intrauterine	Oral	Patient experienced melasma with oral contraceptive pills	Y	N	0	0	0	0	Intrauterine	Oral
24	None	None	None	Y	N	0	0	0	0	Injectable	Transdermal
25	None	None	None	Y	N	0	0	0	0	Implant	Injectable
26	Intravaginal	Implant	Prolonged bleeding after implant placement	Y	N	0	0	0	0	Intravaginal	Implant
27	Oral	Transdermal	Patient felt nauseated and the patch fell off on day 3 or 4	Y	N	1	0	0	0	Intravaginal	Oral
28	None	None	None	Y	N	0	0	0	0	Transdermal	Intravaginal

29	Oral	Intravaginal	Not specified in the clinical note	Y	N	0	0	0	0	Oral	Intravaginal
30	None	Implant	The implant was removed,specific reason not stated in the note.	Y	N	0	0	0	0	Oral	Implant
31	None	None	None	Y	N	0	0	0	0	Intrauterine	Oral
32	Intravaginal	Intrauterine	Patient had no periods since insertion and found insertion process painful.	Y	N	0	0	0	0	Intravaginal	Oral
33	Oral	Intravaginal	Not helping with acne,patient wants to try a different method	Y	N	1	0	0	0	Oral	Intravaginal
34	Implant	None	None	Y	N	0	0	0	0	Implant	Oral
35	Oral	Implant	Patient has been bleeding more frequently	Y	N	0	0	0	0	Oral	Implant
36	None	None	None	Y	N	0	0	0	0	Intravaginal	Injectable
37	Transdermal	None	None	Y	N	1	0	0	0	Transdermal	Intravaginal
38	Intrauterine	None	Patient desired a more long-term method of contraception	N	Y	0	0	0	0	Intrauterine	Oral
39	Injectable	None	None	Y	N	1	0	0	0	Injectable	Oral
40	None	Implant	Patient requested removal and is interested in tubal ligation.	Y	N	0	0	0	0	Injectable	Oral
41	Implant	None	None	Y	N	0	0	0	0	Implant	Injectable
42	None	None	None	Y	N	0	0	0	0	Intravaginal	Oral
43	None	None	None	Y	N	0	0	0	0	Injectable	Oral
44	None	None	None	Y	N	0	0	0	0	Transdermal	Oral
45	Transdermal	Transdermal	Patient's preference and risk of reinfection from untreated partner.	N	N	0	1	0	0	Transdermal	Implant
46	Intravaginal	None	None	Y	N	0	0	0	0	Intravaginal	Oral
47	None	None	None	Y	N	0	0	0	0	Oral	Transdermal
48	None	None	None	Y	N	0	0	0	0	Oral	Intravaginal
49	Intrauterine	None	None	Y	N	0	0	0	0	Intrauterine	Implant
50	Implant	None	None	Y	N	0	0	0	0	Implant	Oral
51	Oral	None	None	Y	N	0	0	0	1	Oral	Implant
52	Oral	Intrauterine	Patient experiencing vaginal dryness and discharge.	Y	N	1	1	0	0	Oral	Intrauterine
53	None	None	None	Y	N	0	0	0	0	Oral	Injectable
54	None	None	Patient was unable to tolerate oral contraceptive pill.	N	Y	0	0	0	0	Transdermal	Oral
55	Intravaginal	Oral	Stopped due to mood changes,tried multiple pill types	Y	N	0	0	0	0	Intravaginal	Oral
56	Implant	Oral	She had irritability and decreased libido on Depo.	N	N	0	1	0	0	Implant	Transdermal
57	Implant	None	Patient is contemplating a 3rd child and needed a new contraceptive method	N	N	1	0	0	0	Implant	Intrauterine
58	Implant	None	None	Y	N	0	0	0	0	Implant	Intravaginal
59	None	None	None	Y	N	0	0	0	0	Oral	Implant
60	None	None	None	Y	N	0	0	0	0	Intravaginal	Oral
61	Oral	Intrauterine	IUD partially extruded from cervix causing potential complications	Y	N	0	0	0	0	Oral	Intrauterine

62	None	Injectable	Persistent vaginal bleeding and patient's discomfort with birth control	Y	N	0	0	0	0	Oral	Injectable
63	Transdermal	Transdermal	Patient didn't want to take it but also doesn't want to get pregnant.	Y	N	0	0	0	0	Transdermal	Oral
64	Intravaginal	Implant	Mood changes,abnormal uterine bleeding,and numbness in hands and legs	Y	N	0	0	0	0	Intravaginal	Implant
65	None	Intrauterine	IUD malpositioned with arm extending into right endometrium	Y	N	0	0	1	0	Implant	Intravaginal
66	Implant	Oral	None	Y	N	0	0	0	0	Implant	Oral
67	Transdermal	Implant	Patient experienced spotting issues with the implant.	Y	N	0	0	0	0	Transdermal	Oral
68	None	None	None	Y	N	0	0	0	0	Intravaginal	Oral
69	Implant	Oral	None	Y	N	0	1	0	0	Implant	Transdermal
70	None	None	None	Y	N	0	0	0	0	Oral	Transdermal
71	Implant	None	None	Y	N	0	0	0	0	Implant	Transdermal
72	Oral	Implant	Patient was gaining weight and desired a different method	Y	N	1	0	0	0	Oral	Implant
73	Injectable	Intravaginal	Patient kept forgetting to remove & re-insert on schedule	Y	N	0	0	0	0	Injectable	Intravaginal
74	Intrauterine	None	None	Y	N	0	0	0	0	Intrauterine	Oral
75	None	None	None	Y	N	0	0	0	0	Implant	Transdermal
76	Intrauterine	None	None	Y	N	0	0	0	1	Intrauterine	Implant
77	Oral	Implant	Patient had irregular menses and preferred to switch methods.	Y	N	0	0	0	0	Oral	Implant
78	Intrauterine	None	Patient desired IUD insertion for management of bleeding.	Y	N	0	0	0	0	Intrauterine	Intravaginal
79	None	None	None	Y	N	0	0	0	0	Implant	Injectable
80	Injectable	Oral	Patient interested in depo,irregular menses despite oral contraceptive use	N	N	0	0	0	0	Injectable	Oral
81	Oral	None	None	Y	N	0	0	0	0	Oral	Injectable
82	Intrauterine	None	None	Y	N	0	0	0	0	Intrauterine	Oral
83	None	None	None	Y	N	0	0	0	0	Intravaginal	Transdermal
84	Implant	None	None	Y	N	0	0	0	0	Implant	Oral
85	Intrauterine	None	None	Y	N	0	0	0	0	Intrauterine	Oral
86	Transdermal	None	Patient experienced irregular heavy menstrual bleeding and low libido.	Y	N	0	0	0	1	Transdermal	Implant
87	Transdermal	None	Patient has a hard time remembering pills	N	N	0	0	0	0	Transdermal	Oral
88	Implant	None	None	Y	N	0	0	0	0	Implant	Injectable
89	Injectable	None	None	Y	N	0	0	0	0	Injectable	Oral
90	Injectable	None	None	Y	N	0	0	0	0	Injectable	Oral
91	None	None	None	Y	N	0	0	0	0	Intrauterine	Oral
92	Intrauterine	Oral	Patient reported not taking it on <REDACTED DATE>	N	N	1	1	0	0	Implant	Oral

Supplementary Table 6: BERTopic clustering and manual grouping of reasons for contraceptive switching.
BERTopic was used to cluster reasons for contraceptive switching extracted by GPT4. Topic clusters were manually reviewed and similar topics were aggregated.

**Original Topic**	**Original Topic Top Terms**	**New Topic**	New Topic Top Terms
0	None	0	None
1	bleeding,experienced,method,effects,side,desired,spotting,weight,breakthrough,control	1	bleeding,experience,spotting,irregular,method,breakthrough,experiencing,weight
2	bleeding,spotting,irregular,ongoing,experiencing,daily,continued,prolonged,breakthrough,experienced	1	bleeding,experience,spotting,irregular,method,breakthrough,experiencing,weight
3	take,remembering,forgets,pills,difficulty,oral,often,pill,want,time	2	iud,contraceptive,bleeding,desired,switch,insertion,oral,experienced
4	take,remembering,symptoms,pills,patch,pill,stopped,may,year,trouble	2	iud,contraceptive,bleeding,desired,switch,insertion,oral,experienced
5	method,desired,contraceptive,oral,switch,free,contraception,regulate,irregular,patch	3	take,remembering,pills,forgets,pill,difficulty,oral,daily
6	increased,irritation,experienced,implant,intake,arm,mirena,pain,expired,complained	4	experienced,irritation,implant,rash,nuvaring,increased,taking,complained
7	iud,partner,removed,malpositioned,could,partially,feel,removal,cervix,uterus	5	iud,removed,malpositioned,partner,device,intrauterine,could,partially
8	contraceptive,oral,wanted,pills,transition,copper,method,switching,decided,switch	3	take,remembering,pills,forgets,pill,difficulty,oral,daily
9	shot,depo,permanent,missed,bleeding,breakthrough,ocps,provera,weight,method	1	bleeding,experience,spotting,irregular,method,breakthrough,experiencing,weight
10	irritability,mood,gain,weight,tolerating,swings,decreased,vomiting,discontinuation,hair	6	gain,weight,irritability,tolerating,mood,increased,decreased,vomiting
11	irregular,menses,bleeding,discharge,vaginal,dysmenorrhea,current,experiencing,intercourse,pain	7	irregular,vaginal,bleeding,menses,intercourse,discharge,pain,experiencing
12	rahs,stopped,skin,taking,episodes,allergic,facial,swelling,showers,took	4	experienced,irritation,implant,rash,nuvaring,increased,taking,complained
13	control,birth,wants,options,disucss,vacation,medical,nuva,form,considering	3	take,remembering,pills,forgets,pill,difficulty,oral,daily
14	pregnancy,termination,alternate,desires,therapy,wishes,simpler,regimen,presented,discontinue	3	take,remembering,pills,forgets,pill,difficulty,oral,daily
15	ocp,cover,insurance,ring,ocps,patch,requested,worries,coming,evra	8	patch,ocp,cover,take,forgetting,insurance,ring,ocps
16	implant,removed,removal,placement,proceed,reinserted,expiration,desired,fibroids,site	9	implant,removed,removal,placement,desired,proceed,reinserted,expiration
17	insertion,contraception,desired,iud,switch,opted,change	3	take,remembering,pills,forgets,pill,difficulty,oral,daily
18	nuvaring,expressed,ovarian,desire,swings,ocps,understand,function,placebo,became	3	take,remembering,pills,forgets,pill,difficulty,oral,daily

Supplementary Table 7: Prevalence of topics related to contraceptive switching extracted using BERTopic.
The number of clinical notes assigned to each topic identified using BERTopic applied to reasons for contraceptive switching extracted using GPT4.

Topic ID	Count	Topic Top Terms	Assigned Topic Name
0	1136	0_none__	NA
1	272	1_bleeding_experienced_spotting	Irregular bleeding
2	267	2_iud_contraceptive_bleeding	Patient preference
3	116	3_take_remembering_pills	Forgetting daily pills
4	68	4_experienced_irritation_implant	Irritation/Rash
5	31	5_iud_removed_malpositioned	IUD malpositioned
6	21	6_gain_weight_irritability	Weight gain & mood changes
7	21	7_irregular_vaginal_bleeding	Irregular menses & pain
8	20	8_patch_ocp_cover	Insurance coverage
9	12	9_implant_removed_removal	Implant removal

Supplementary Table 8: Evaluation of open source models on validation dataset using labels from structured data and versus expert annotation. Bolded values represent the highest scoring model for each label type.

The performance of 8 open source language models on contraceptive information extraction was also tested using the specialist system message and default prompt. Greedy sampling, 8-bit quantization, and a maximum response length of 250 was used for all open-source models. Extracted information for medication start and stop information were assessed relative to structured data as well as gold-standard clinical annotations. Of these models, Gemma2-9B-it showed the best performance with the highest microF1 scores for both medication start (0.806) and stop (0.882) extraction when compared to expert annotation.

Label	Model	MicroF1 (vs Structured data)	MicroF1 (vs Expert Annotation)
Medication Started	gemma2-9b-it	0.848	0.806
	Meta-Llama-3.1-8B-Instruct	0.783	0.806
	JSL-MedMNX-7B-SFT	0.797	0.774
	starling-7b-alpha	0.768	0.763
	gemma-7b-it	0.702	0.677
	starling-7b-beta	0.609	0.645
	llama-3-8b-chat-hf	0.472	0.581
	BioMistral-7B	0.705	0.548
Medication Stopped	gemma2-9b-it	0.403	0.882
	starling-7b-alpha	0.352	0.849
	JSL-MedMNX-7B-SFT	0.409	0.839
	Meta-Llama-3.1-8B-Instruct	0.373	0.839
	gemma-7b-it	0.168	0.782
	starling-7b-beta	0.261	0.720
	llama-3-8b-chat-hf	0.173	0.710
	BioMistral-7B	0.386	0.612
